# Electrically Switchable Multi‐Stable Topological States Enabled by Surface‐Induced Frustration in Nematic Liquid Crystal Cells

**DOI:** 10.1002/adma.202414675

**Published:** 2025-01-10

**Authors:** Jelto Neirynck, Yu‐Tung Hsiao, Migle Stebryte, Inge Nys

**Affiliations:** ^1^ Liquid Crystals and Photonics Group Department of Electronics and Information Systems Ghent University Technologiepark‐Zwijnaarde 126 Ghent 9052 Belgium

**Keywords:** disclination lines, dual‐frequency nematic liquid crystals, electrical switching, multi‐stability, photoalignment patterning

## Abstract

In liquid crystal (LC) cells, the surface patterning directs the self‐assembly of the uniaxial building blocks in the bulk, enabling the design of stimuli‐response optical devices with various functionalities. The combination of different anchoring patterns at both substrates can lead to surface induced frustration, preventing a purely planar and defect‐free configuration. In cells with crossed assembly of rotating anchoring patterns, elastic deformations allow to obtain a defect‐free bulk configuration, but an electrical stimulus can induce disclination lines. The disclination network is preserved without applied voltage. Depending on the electric field treatment and geometrical parameters, different multi‐stable states with and without disclinations are obtained. This is demonstrated with the help of dual‐frequency LCs, for which the frequency dependent dielectric properties allow repeatable switching between multi‐stable states. Topological protection and the associated energy barrier between different states explains the observed metastability. The obtained configurations are retrieved with Q‐tensor simulations and the validity is confirmed by matching optical simulations with experimentally obtained microscopy images. The realized multi‐stable topological states interact differently with light, resulting in distinct optical properties. Optimization allows to switch between a highly transparent state and an opaque state, opening up opportunities for smart windows with low energy consumption.

## Graphical abstract

In a liquid crystal (LC) cell with well‐designed surface anchoring patterns, electrical switching between multi‐stable states with distinct optical properties is obtained. Dual‐frequency LC with frequency dependent dielectric properties is used to realize repeatable switching between different topological states with and without disclination lines. Metastability without the need for an applied voltage is demonstrated.

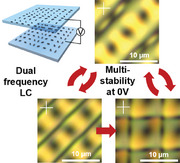

## 1 Introduction

Liquid crystalline materials, that combine fluidity with long‐range ordering of their anisotropic building blocks, offer an interesting playground to develop stimuli‐responsive devices and to study fundamental properties of topological defect formation. Nematic liquid crystals (LCs), in which the rod‐shaped molecules possess orientational ordering but no positional ordering, are most studied thanks to their successful use in several applications, including displays,^[^ ^1^, ^2^, ^3^, ^4^ ^]^ geometric phase devices,^[^ ^5^, ^6^, ^7^, ^8^ ^]^ sensors,^[^ ^9^, ^10^ ^]^ anticounterfeiting labels^[^ ^11^, ^12^ ^]^ and soft robots.^[^ ^13^, ^14^ ^]^ For traditional electro‐optic applications such as LC displays, a defect‐free LC director configuration is usually aimed for. However, topological defects are ubiquitous in nature (in several condensed matter systems,^[^ ^15^, ^16^, ^17^ ^]^ in acoustic and optics,^[^ ^18^ ^]^ in particle physics,^[^ ^19^ ^]^ in the early universe,^[^ ^20^ ^]^ etc.) and their presence usually strongly affects the physical behavior of a material system. Topological defects in LCs, in which the ordering is locally frustrated due to topological reasons, are named “disclinations”. Nowadays fundamental studies on LC disclinations are complemented by more applied studies in which disclination lines are used to enable novel applications. The possibility to control and rewire disclination lines is therefore often sought for.^[^ ^21^, ^22^, ^23^, ^24^, ^25^ ^]^ Disclination lines have been used to steer colloidal assembly, for micro‐transport, to induce drastic shape‐deformations in LC elastomers and for electro‐optic applications (optical vortex generation, tunable gratings, scattering states, etc.).^[^ ^26^, ^27^, ^28^, ^29^ ^]^ The topological defects exhibit a unique optical signature,^[^ ^27^ ^]^ affect the rheological properties of the LC, can trap colloidal objects,^[^ ^28^ ^]^ segregate impurities,^[^ ^29^ ^]^ etc. Regular grids of LC disclination lines or defects (with well‐controlled periodicity in one or 2D) are also relevant for electro‐optical applications, with well‐defined diffraction patterns appearing due to interaction of a light beam with a regular defect array. The resulting optical properties depend on the type of defects, the symmetry of the pattern, the exact director configuration, the LC birefringence, the layer thickness, etc. A detailed investigation of the diffraction properties has previously been reported mainly for umbilic defect arrays, created in negative dielectric anisotropy LC cells doped with ionic impurities and treated with an electrically resistive alignment layer inducing homeotropic anchoring.^[^ ^30^, ^31^, ^32^, ^33^ ^]^ The umbilical defect arrays are introduced by alternating current (AC) voltage application and the properties depend on the amplitude and the frequency of the applied voltage. Moreover, the symmetry and periodicity can be controlled with the help of micropillar arrays, offering additional control over the diffraction properties. The defect arrays in this material system are induced by voltage application, but other techniques allow to stabilize LC defect configurations without the need for a continuous voltage application.

A standard method to induce disclination lines in a LC cell, without the need for voltage application, uses surface anchoring patterns including defect points.^[^ ^23^, ^25^, ^34^, ^35^, ^36^ ^]^ By imposing defect points at one or both confining substrates, bulk disclination lines are naturally created, connecting surface defect points that are in close proximity. Relaxation to a defect‐free configuration is prevented by the presence of defect points in the surface anchoring. These kind of surface anchoring patterns have been realized with the help of atomic force microscope scribing^[^ ^25^ ^]^ and photoalignment patterning.^[^ ^23^, ^34^, ^35^, ^36^ ^]^ Photoalignment patterning is an industrially relevant technique that allows to precisely engineer the bulk LC configuration by imposing anchoring patterns with submicrometer feature sizes.^[^ ^8^, ^37^, ^38^, ^39^, ^40^ ^]^ Making use of a photo‐sensitive alignment layer, close to arbitrary anchoring patterns can be imposed at the substrates and different alignment patterns at both confining substrates can be combined. Judicious design of the device configuration allows to realize a multitude of highly efficient flat optical components^[^ ^5^, ^6^, ^7^, ^8^, ^41^, ^42^ ^]^ and enables the creation of well controlled disclination networks^[^ ^23^, ^34^, ^35^, ^36^ ^]^ and topologically protected states.^[^ ^43^, ^44^, ^45^ ^]^

Apart from defect patterning at the surfaces, an alternative method to create disclination lines in photopatterned LC cells, makes use of rotated assembly of 2 periodically rotating planar anchoring patterns at the top and bottom substrate.^[^ ^46^, ^47^ ^]^ The surface anchoring patterns are defect‐free in these configurations (with a periodic rotation of the azimuthal anchoring angle), but sliding substrate assembly has been shown to induce disclination lines in the bulk of the layer.^[^ ^44^, ^45^ ^]^ X. Wang et al. recently demonstrated that depending on the alignment period, cell thickness and rotation angle between the 2 substrates, different defect structures can be achieved.^[^ ^46^ ^]^ They examined so‐called “Moire patterns”, for which the angle between the top and bottom substrates is small (below 50°). For cells that are thin compared to the alignment period, the anchoring strength dominates and a pattern of equidistantly spaced straight disclination lines is achieved (resolving the twist conflict at locations with a 90° anchoring mismatch). For intermediate cell thicknesses, the existence of a helical defect structure was described and for thick cells (compared to the period), a grid or web‐like pattern of disclination lines was achieved. A similar web‐like disclination pattern was also achieved for a perpendicular rotation angle between both substrates by M. Wang et al.,^[^ ^47^ ^]^ by using sliding assembly of 2 square surface alignment patterns (in the presence of LC).

**Figure 1:**
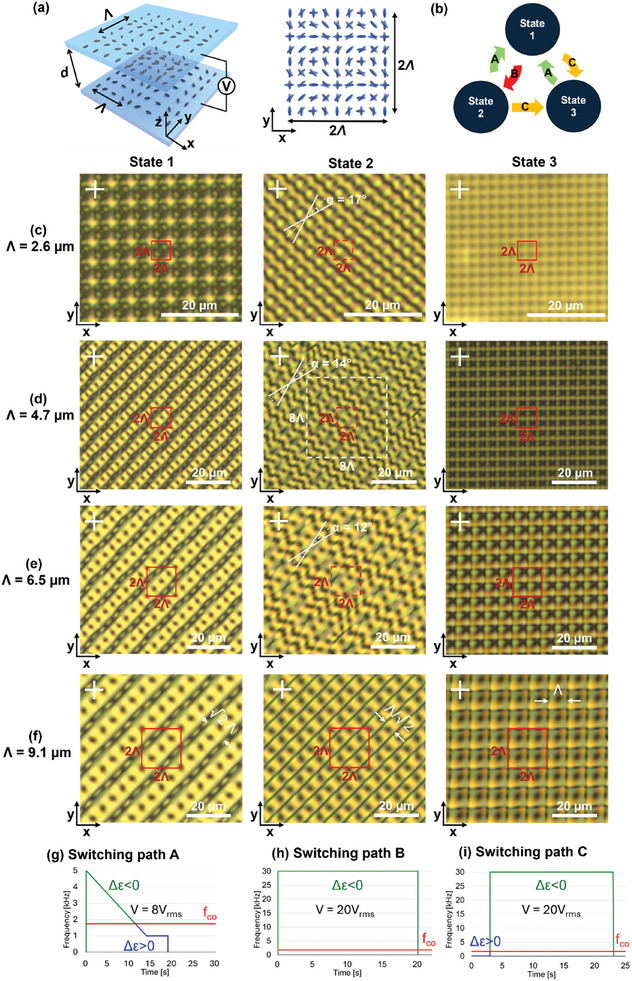
a) Schematic representation of the surface anchoring conditions imposed by photoalignment patterning; b) Switching paths between the 3 different stable states obtained at 0V; c–f) Polarizing optical microscopy image of state 1 (left), state 2 (middle) and state 3 (right) between crossed polarizers for alignment period *Λ* = 2.6 µm (c), *Λ* = 4.7 µm (d), *Λ* = 6.5 µm (e) and *Λ* = 9.1 µm (f). The red square indicates a 2*Λ* by 2*Λ* area (corresponding with the area of the simulated unit cell in Figure 3). The cell thickness is *d* = 3.5 µm. POM images of a larger area, showing some imperfections, are shown in Figure S1 (Supporting Information). g–i) Frequency and voltage paths to reach each state. The green (blue) line indicates frequencies for which the material behaves negative (positive) anisotropic. The red line indicates the cross‐over frequency. Path A has a constant voltage amplitude of 8 V_rms_ while path B and C use a constant voltage of 20 V_rms_. The left part of Figure 1a is reused with small modification from Figure 1 in reference.^[^ ^52^ ^]^

The rotated assembly of 2 substrates with a periodically rotating anchoring pattern, as shown in **Figure** **1**a), leads to surface induced frustration. The anchoring constraints prevent a purely flat and defect‐free LC orientation with only twist deformations between the confining substrates. As a result, sliding substrate assembly can lead to the creation of disclination lines.^[^ ^46^, ^47^ ^]^ However, as we demonstrated previously, usual splay, twist and bend deformations in the LC allow to resolve the frustration imposed by the anchoring constraints without the need for singular disclination lines.^[^ ^48^, ^49^, ^50^, ^51^ ^]^ To do so, the director locally tilts out‐of‐plane, creating regions with vertical director orientation in the bulk of the layer. The symmetry breaking that is required to induce a defect‐free bulk configuration, leads to the formation of a unit cell (2*Λ* × 2*Λ*) that is 4 times larger than the unit cell of the surface anchoring pattern (*Λ* × *Λ*). In devices with fixed anchoring conditions (without moving elements), this defect‐free configuration has been shown to be the most prevalent in devices with a large range of alignment periods and cell thicknesses.^[^ ^48^, ^49^, ^50^, ^51^ ^]^

So far, these type of devices have only been studied for nematic LC with a positive dielectric anisotropy. Field‐induced tuning of the optical properties (resulting from a smooth reorientation toward a vertical aligned state) can be seen in non‐chiral LC cells,^[^ ^48^, ^49^, ^50^ ^]^ and some hysteresis behavior, with switching between 2 different topological states, has been demonstrated in cells filled with long‐pitch chiral LC.^[^ ^52^ ^]^ The addition of chirality to the LC allows to induce a novel topological state at low voltages, enabling hysteretic behavior, but the control over the resulting optical properties has been limited and the realization of multi‐stability without the need for an applied voltage has been elusive.

To overcome these limitations and enable novel configurations with advanced switchability, we here make use of the frequency dependent dielectric properties of a (non‐chiral) dual‐frequency liquid crystal (DFLC). By using DFLC in a cell with crossed assembly of rotating anchoring patterns (Figure 1a), switching between a defect‐free director configuration and 2 defect‐containing states is realized. A topological barrier and energy barrier exists between the 3 different configurations, explaining the observed multi‐stability without the need for an applied voltage. To fully understand the experimentally obtained results (as discussed in section 2), finite element (FE) Q‐tensor simulations are used in combination with optical simulations for the near‐field microscopy images and the far‐field diffraction patterns (section 3). We focus on the stabilization of different topological states without the need for an externally applied voltage, and explain the origin of the obtained metastable configurations (section 4). Switching paths between the different states are unveiled and the effect of variations in the geometrical parameters (cell thickness, alignment period) is analyzed. Moreover, the robustness of the proposed device configuration is further confirmed by using another type of DFLC material and testing different cell thicknesses. To elucidate the application potential of the presented work, the optical properties of the realized multi‐stable states are qualitatively and quantitatively analyzed, confirming that device optimization allows to realize multi‐stable states with very distinct optical properties.

## 2 Experimental Results

### 2.1 Polarizing Optical Microscopy Images

The experimental results presented in Figure 1 demonstrate that 3 different multi‐stable bulk configurations can be realized by using an appropriate electric field treatment in a cell with crossed assembly of periodically rotating anchoring patterns at the top and bottom substrate (Figure 1a). Multi‐stability in the absence of an externally applied electric field is enabled by using dual‐frequency LC (DFLC 1952H, prepared at Warsaw Military University, Poland, see Materials and Methods). Polarizing optical microscopy (POM) images of the experimentally obtained metastable states at 0V are shown in Figure 1c–f for a cell with thickness *d* = 3.5 µm and 4 different surface alignment periods *Λ* = 2.6 µm (c), 4.7 µm (d), 6.5 µm (e) and 9.1 µm (f) over which the director is rotating 180°. All 3 topological states are stable after the removal of the voltage, but dedicated switching paths exist to switch reliably and repeatably between the different states (Figure 1b,g–i).

After filling the cell at elevated temperature (90 °C) and cooling down to room temperature, state 1 is obtained in the entire photopatterned area with a limited amount of defects (Figure S1, Supporting Information). Different domains exist, shifted over a period *Λ* in the horizontal and or vertical direction, separated by a defect line (Figure S1, Supporting Information). However, inside one domain a defect‐free configuration is obtained. POM images show a diagonal texture with an alteration of 2 different regions: one region that appears bright but is periodically interrupted with black dots (with period *Λ*/√2 along the diagonal) and one region that is darker and that could be perceived as 2 intertwining wobbling black lines between crossed polarizers. For all but the smallest alignment period (*Λ* = 2.6 µm) the observed texture in POM looks very similar, with the wobbling black line region spreading over a relatively larger area for decreasing alignment periods. For the smallest alignment period of *Λ* = 2.6 µm the structure is deformed and is no longer recognized as a diagonal texture in POM.

Starting from this first configuration (state 1), state 2 can be reached by applying a voltage (20 V_rms_) in the negative dielectric anisotropy range (*f* = 30 kHz, path B as shown in Figure 1h). As seen in Video S6 (Supplementary Video) in the supporting information, the darker black band with 2 intertwining lines in POM for state 1, splits into 2 thin black lines that appear to be disclination lines (as will be confirmed later on). As a result, for the largest alignment period *Λ* = 9.1 µm, the POM image between crossed polarizers shows a bright background with a periodic grid of black dots (with period *Λ*/√ 2 along the diagonal and anti‐diagonal), superimposed by a periodic array of thin black disclination lines that are oriented parallel to the diagonal and separated by *Λ*/√2. For smaller alignment periods *Λ *= 2.6, 4.7, and 6.5 µm deviations appear from this grid with diagonal black lines: the lines are no longer running parallel to the diagonal but a grid is formed with 2 sets of disclination lines, oriented respectively over 2 angles 45° + α and 45° – α that are symmetric with respect to the diagonal. We call this the “deformed state 2” since it is reached with the same switching procedure as state 2, starting from state 1 and applying a voltage in the negative dielectric anisotropy range. The smaller the surface alignment period, the larger the value for α becomes, leading to a larger intersection angle of the disclination lines and a smaller dimension of the periodic unit cell that is formed. As demonstrated in Figures S1 and S3 (Supporting Information) the periodicity of this structure breaks down somewhat over large areas, with deviations in the orientation angle of the disclination lines showing up over larger distances (tens of alignment periods). For decreasing alignment period *Λ*, the thin black disclination lines become less and less visible in POM between crossed polarizers. However, POM images without polarizers can more clearly reveal the presence of disclination lines even for small alignment periods, as shown in Figure S3 (Supporting Information).

A different topology is formed in state 3, with thin black disclination lines running along the x‐ and y‐direction, perpendicular to the grating vector at the top and bottom substrate respectively (Figure 1). Both the horizontal and vertical disclination lines (along the x‐ and y‐axis) are separated by *Λ*, creating a *Λ* by *Λ* periodic square grid of disclination lines. Although the disclination lines become increasingly less visible for decreasing alignment periods *Λ*, a square texture is observed in POM for all periods (Figure 1). To reach state 3, switching path C should be followed (Figure 1b). This path includes voltage application (of 20 V_rms_) in the positive dielectric anisotropy range (100 Hz, Δɛ >0) immediately followed by application of a voltage (of 20 V_rms_) in the negative dielectric anisotropy range (30 kHz, Δɛ < 0), as shown in Figure 1i. The same electric field treatment can be used, irrespective of the starting configuration (state 1 or state 2).

Finally, to switch back from (deformed) state 2 or state 3 to state 1 (path A, Figure 1g), an essential step is to apply a voltage in the positive dielectric anisotropy range before turning off the voltage. However, directly applying a voltage with a frequency below the crossover frequency f_co_ (*f* < *f*_co_) does not appear to be a very reliable switching strategy. As can be seen in Video S1 and S2 (Supplementary Video) (with state 3 as a starting configuration), the resulting configuration after removal of the voltage in this case depends on the amplitude of the applied voltage (8 V_rms_ in Video S1, 20 V_rms_ in Video S2) and sometimes the structure partially relaxes back to the initial state 3 instead of creating state 1. This effect is even worse for the DFLC material 1999A, as can be seen in Video S8 (Supplementary Video) (for 8 Vrms) and Video S9 (for 16 Vrms). To obtain a reliable switching method that ensures the creation of a high quality state 1, it beneficial to first destabilize the existing disclination grid (and the associated structural information), before applying a voltage with *f* < *f*_co_. This can effectively be realized by applying a voltage with *f* ≈ *f*_co_ at the beginning of the switching path. As can be seen in Figure 1(g), instead of first applying a voltage with *f *= *f*_co_, we opt to use a variable frequency in the beginning of the switching path, decreasing the frequency from *f* = 5 kHz (*f* > *f*_co)_ to *f* = 1 kHz (*f* < *f*_co_) at a fixed applied voltage (of 8 V_rms_). By doing so, we ensure that the crossover frequency is crossed at a certain moment during the switching path, limiting the sensitivity to the exact working temperature (and therefore to the exact value of the crossover frequency). Voltage application with *f* ≈ *f_co_* gives rise to strong director fluctuations and allows to more easily destabilize the disclination lines in state 2 or state 3 (see Video S3 and Video S10 (Supplementary Video) for 1952H and 1999A respectively). This allows to obtain a more defect‐free state 1 that remains stable after completing the switching path. Different examined switching paths are shown in Videos S1–S5 and S8–S12 (Supplementary Video). Optimization of the switching path becomes more essential for thicker cells and the results are also dependent on the used DFLC material mixture.

Finally, remark that although we are mainly interested in electrical switching paths between the different multi‐stable states, heating of the cell could potentially also be used to switch back to state 1 (starting from any configuration). The thermal switching mechanism is however complicated by the high nematic‐isotropic transition temperature of the used DFLC materials in our cells (141.1 and 154.8 °C respectively for the 1999A and 1952H mixture), and the limited temperature range (<90 °C) the glue (NOA 68) can withstand. Thermal switching was therefore not explored, but we focus on the electrical switching mechanism that is enabled by using dual‐frequency LC material.

### 2.2 Diffraction Measurements

**Figure 2:**
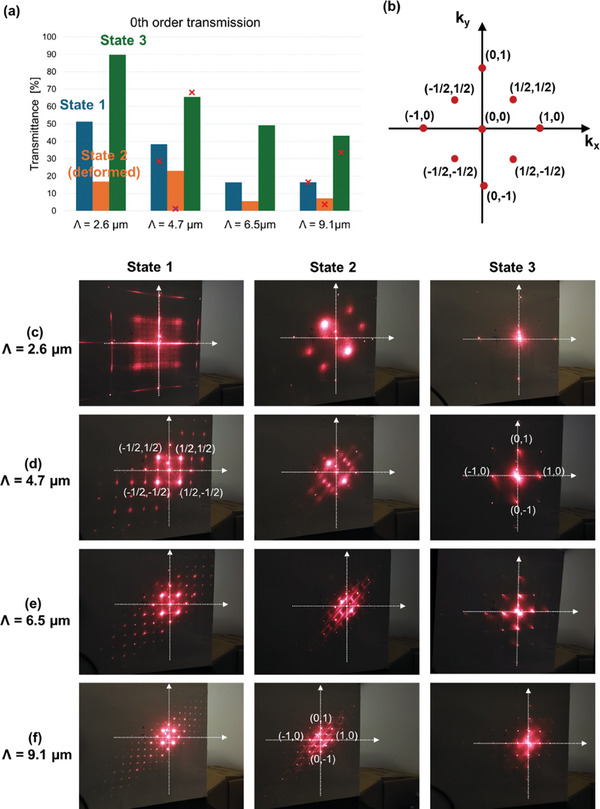
Transmittance of the zeroth diffraction order a) and corresponding diffraction patterns c–f) for the 3 different states in a cell with thickness *d* = 3.5 µm and alignment period *Λ *= 2.6 µm (c), *Λ* = 4.7 µm (d), *Λ* = 6.5 µm (e), *Λ* = 9.1 µm (f). The measurements are performed with a Helium‐Neon laser *λ* = 633 nm for linearly polarized incident light. The k‐vector diagram used for the description of the diffraction orders is represented in b) and the corresponding nomenclature is used to indicate the most important non‐zero diffraction orders in (d). The red crosses in (a) indicate the simulated values corresponding with the diffraction patterns in **Figure** **3** (with the assumption of equal elastic constants) for simulated alignment period *Λ* = 4.5 µm and *Λ* = 9 µm. The purple dot indicates the simulated value for the deformed state 2 (corresponding with Figure 4b) for *Λ* = 4.5 µm.

The 3 different metastable states at 0V have a different appearance in POM (Figure 1) and also give rise to a distinct far‐field diffraction pattern (**Figure** **2**). As a quantitative measure, the 0 order transmission is measured for the 3 different states (Figure 2a) and their respective diffraction patterns are shown for linearly polarized incident light (λ = 633 nm) for the 4 different surface alignment periods *Λ* = 2.6, 4.7, 6.5, and 9.1 µm (Figure 2b–e). Some similarities can be seen for the different alignment periods, showing that state 3 has the highest 0 order transmission while (deformed) state 2 has the lowest 0 order transmission. State 1 has an intermediate behavior.

The diffraction pattern for state 1 (and for the alignment patterns with *Λ* = 4.7, 6.5, and 9.1 µm) shows the central (0 order) transmission, mainly surrounded by 4 diffraction orders on the corners of a square. The diffraction angle for these 4 diffraction orders, denoted as (k_x_, k_y_) = (+−1/2, +−1/2) in Figure 2, is $\pm \mathrm{asin}(\frac{\lambda }{\surd 2\Lambda })$ along the diagonal or anti‐diagonal direction respectively. Also the somewhat weaker (k_x_, k_y_) = (+−1, 0) and (k_x_, k_y_) = (0, +−1) diffraction orders can be recognized in the diffraction pattern.

The diffraction pattern of (deformed) state 2 is mirror symmetric with respect to the diagonal, and for the smallest alignment periods the 2 dominant diffraction orders are situated along the anti‐diagonal. For the larger alignment periods (and especially for *Λ* = 9.1 µm), relatively strong diffraction is also observed in the horizontal and vertical direction, corresponding to orders (k_x_, k_y_) = (0, 1), (1,0), (0,−1) and (−1,0), and in the diagonal orders (k_x_, k_y_) = (1,1) and (−1,−1).

The diffraction pattern for state 3 essentially shows a dominant 0 order transmission, combined with the same 4 diffraction orders along the horizontal and vertical direction (k_x_, k_y_) = (0, 1), (1,0), (0,−1) and (−1,0). Especially for the smallest alignment period *Λ* = 2.6 µm, a remarkably high amount of light is transmitted in the 0^th^ order.

## 3 Simulations

**Figure 3:**
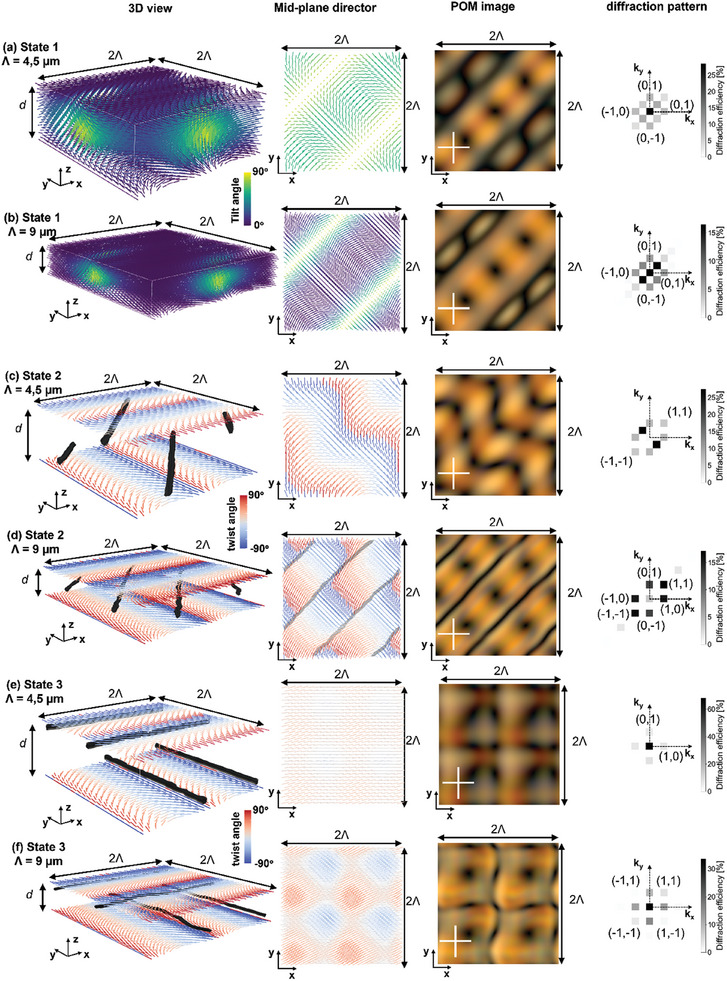
Simulated metastable director configurations without applied voltage in a cell with thickness *d* = 3.5 µm for alignment period *Λ* = 4.5 µm (a,c,e) and *Λ* = 9 µm (b,d,f). The results for state 1 a,b), state 2 c,d) and state 3 e,f) are shown from left to right: a 3D view, the mid‐plane cross‐section, the simulated POM image and the simulated diffraction pattern for red light *λ* = 633 nm. For state 1, the color bar represents the tilt‐angle with respect to the xy‐plane, for state 2 and 3 the color bar represents the twist angle with respect to the x‐axis. Regions with a reduced order parameter (equal to 0.35) for state 2 and 3 are shown in grey, representing the disclination lines. In the mid‐plane cross‐section (second column), the disclination lines are only presented when they lie in this plane (for state 2 with *Λ* = 9 µm).

To understand the origin of the experimentally observed multi‐stable states, finite element (FE) Q‐tensor simulations are performed. The resulting director configurations are used as input for the optical simulations that predict the polarizing optical microscopy images and far field diffraction patterns (see methods section). The simulation results for the director configuration corresponding to state 1, 2 and 3 are summarized in Figure 3. The results are shown for cell thickness *d* = 3.5 µm and 2 different alignment periods *Λ* = 4.5 µm and *Λ* = 9 µm.

The simulations for state 1 show a defect‐free director configuration with localized regions with a close to vertical mid‐plane director orientation (Figure 3a,b). These are running parallel to the diagonal (x = y) and neighboring regions with vertical director orientation are spaced by √2*Λ* along the anti‐diagonal. In between regions with a vertical mid‐plane director orientation, areas exist with a close to planar mid‐plane director that is periodically rotating along the diagonal direction with period √2*Λ*. This rotation follows the alignment rotation imposed by the surface anchoring patterns at the top and bottom substrate (Figure 1a). For the largest alignment period (*Λ* = 9 µm, Figure 3b) the director is almost perfectly planar in these regions while for the smaller alignment period a more pronounced tilt is present (*Λ* = 4.5 µm, Figure 3a). The corresponding simulated POM images between crossed polarizers show a bright yellow background combined with 2 regions with a different appearance: one bright area is periodically interrupted with black dots (with period *Λ*/√2 along the diagonal) while the other is darker and shows 2 intertwining winding black lines in the bright surrounding. The winding black lines appear with a period √2*Λ* along the anti‐diagonal and correspond with regions with a close to vertical mid‐plane director orientation. The black dots in POM appear in regions with a close to planar bulk director orientation parallel to the analyzer or the polarizer. The simulated diffraction pattern for linearly polarized incident light shows dominant diffraction in the 0 order, in the (k_x_, k_y_) = (+−1, 0) and (k_x_, k_y_) = (0, +−1) orders and in the (k_x_, k_y_) = (+−1/2, +−1/2) orders.

The simulations for state 2 show the presence of singular disclination lines running parallel to the diagonal (x = y) and spaced over a distance *Λ*/√2 along the anti‐diagonal (Figure 3c,d). The disclination lines are represented by regions with a reduced order parameter in Figure 3. For alignment period *Λ* = 9 µm and cell thickness *d* = 3.5 µm, the disclination lines are positioned in the mid‐plane of the cell. In between the disclination lines, the director orientation is planar (parallel to the xy‐plane) and periodically rotating along the diagonal (x = y) with period √2*Λ* (Figure 3d). Deviations appear for the smaller alignment period *Λ* = 4.5 µm, resulting in neighboring disclination lines being positioned respectively closer to the top or bottom substrate (Figure 3c). Experimentally, the ideal state 2 is however not retrieved in cells with a small surface alignment period (see Figure 1c–e), leading to the simulation of a so called “deformed state 2”, as will be discussed in the next paragraph. The simulated POM images for state 2 for *Λ* = 9 µm (Figure 3d) show a bright yellow background combined with thin black lines parallel to the diagonal (spaced by *Λ*/√2) and black dots spaced by a distance *Λ*/√2 along the diagonal and anti‐diagonal. The thin black lines in POM in this case are the signature of the disclination lines. For the smaller alignment period *Λ* = 4.5 µm, the simulations predict that the disclination lines can no longer be clearly recognized in POM images between crossed polarizers (Figure 3c). The simulated diffraction patterns are mirror symmetric with respect to the k_x_ = k_y_ diagonal and the diffraction pattern strongly depends on the simulated alignment period: apart from the 0 order transmission, the (k_x_, k_y_) = (+1/2, −1/2) and (−1/2,+1/2) orders are dominant for the smaller alignment period *Λ* = 4.5 µm while the (k_x_, k_y_) = (1,0), (0,1), (−1,0), (0,−1), (1,1) and (−1,−1) orders become dominant for the larger alignment period *Λ* = 9 µm.

**Figure 4:**
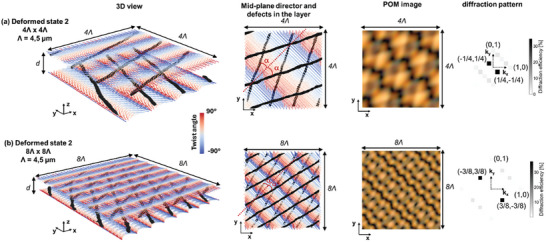
Simulated metastable director configurations without applied voltage for the so‐called deformed state 2 in a cell with thickness *d* = 3.5 µm and alignment period *Λ* = 4.5 µm. A 4*Λ* × 4*Λ* unit cell is simulated in a) while a 8*Λ* × 8*Λ* unit cell is simulated in b). From left to right a 3D view, the mid‐plane cross‐section, the simulated POM image and the simulated diffraction pattern for red light λ = 633 nm are shown. The color bar represents the twist angle with respect to the x‐axis. Regions with a reduced order parameter (equal to 0.35) are shown in grey, representing the disclination lines. For clarity, the disclination lines are included together with the mid‐plane cross‐section for the director (second column), to indicate the orientation angles. The disclination lines lie closer to the top and bottom substrate and are respectively oriented over an angle 45° – α and 45° + α with respect to the x‐axis, with α = 27° in (a) and equal to α = 14° in (b). In the non‐deformed state 2, α = 0° and the rotation speed of the mid‐plane director along the diagonal is π/√2*Λ*, while it is π/2√2*Λ* in (a) and 3π/4√2*Λ* in (b).

The experimentally observed deviations from the ideal state 2 configuration with diagonal disclination lines in cells with small surface alignment period, motivated the performance of additional simulations with an adjusted unit cell dimension (see methods section). Two simulation results for the so called “deformed state 2” are shown in **Figure** 4 for a unit cell with respectively 4*Λ* × 4*Λ* and 8*Λ* × 8*Λ* dimension. Periodic boundary conditions are imposed at the lateral borders of the unit cell and these 2D are chosen to identify the origin of the observed deformation in state 2. The disclination lines, presented by regions with reduced order parameter, are no longer oriented along the diagonal but the lines closer to the bottom or respectively top substrate make an angle 45° ± α with respect to the x‐axis. The angle α is equal to 45°‐ atan(1/3) ≈ 27° in the simulation represented in Figure 4a (with the 4*Λ* × 4*Λ* dimension of the unit cell) while α is equal to 45°‐ atan(3/5) ≈ 14° in the simulation represented in Figure 4b (with the 8*Λ* × 8*Λ* dimension of the unit cell). The orientation of the disclination lines is linked to the mid‐plane director configuration, respectively showing a 2π rotation along the diagonal with 4√2*Λ* length in Figure 4a and a 6π rotation along the diagonal with 8√2*Λ* length in Figure 4b. Compared to the non‐deformed state 2 (Figure 3), with a 2π rotation along the diagonal with 2√2*Λ* length, the rotation speed of the mid‐plane director along the × = y diagonal is decreased in the deformed state 2 (the rotation period is increased). In other words, the mid‐plane director in a cell with a deformed state 2 is no longer following the director rotation as imposed by the surface anchoring patterns at the top and bottom substrate (see Figure 1a). The slower the rotation speed of the mid‐plane director along the diagonal, the larger the intersection angle 2α between the 2 sets of disclination lines (closer to the top and bottom substrate respectively). Apart from the weaker 0 order transmission, the dominant diffraction orders that show up in the diffraction pattern are along the anti‐diagonal direction. Remark that simulations for the deformed state 2 are only shown for a 4*Λ* × 4*Λ* and 8*Λ* × 8*Λ* dimension of the periodic unit cell in Figure 4, but other possibilities with an even larger supercell size (e.g., 12*Λ* × 12*Λ* or 16*Λ* × 16*Λ*) could be considered as well. This would allow to simulate smaller deviations from the non‐deformed state 2 (characterized by smaller α), but comes at a high computational cost and only adds limited additional insights in the formation mechanism of the deformed state 2.

The simulations for state 3 show the presence of a square grid of singular disclination lines running parallel to the x‐ and y‐ axis respectively, with a spacing equal to *Λ* (Figure 3e,f). The disclination lines parallel to the x‐axis are located closer to the top substrate while the disclination lines parallel to the y‐axis are located closer to the bottom substrate. The 2 orthogonal sets of disclination lines are separated by a region with close to uniform director alignment in the middle area of the cell. The director orientation in the mid‐plane is planar (parallel to the xy‐plane), but different from state 1 and 2 no rotation along the diagonal direction is observed. The director is rather uniform instead, with only slight modulations around a preferred orientation (roughly along the x‐ axis in Figure 3e,f). The uniformity of the director in the mid‐plane is further increased for decreasing alignment periods, as can be seen by comparing Figure 3e,f. The smaller the alignment period, the closer the disclination lines are positioned with respect to the surface and the larger the region with close to uniform director orientation between the 2 orthogonal sets of disclination lines becomes. For the largest alignment period *Λ* = 9 µm, the simulated POM images show a roughly square *Λ* × *Λ* periodic structure with thin black lines somewhat representing the disclination lines that are present in the director configuration (Figure 3f). For the smaller alignment period *Λ* = 4.5 µm, a square *Λ* × *Λ* periodic texture is still observed but the presence of disclination lines is less visible in the simulated POM images (Figure 3e). In both cases (*Λ* = 4.5 or 9 µm) the central area of the *Λ* × *Λ* periodic square area is black, corresponding with a region with close to uniform bulk director orientation oriented along one of the polarizers in POM (along the x‐axis in Figure 3e,f). The simulated diffraction pattern is dominated by transmission in the 0 order, with some weak diffraction in the (k_x_, k_y_) = (1,0), (0,1), (−1,0) and (0,−1) orders appearing as well. The efficiency of the 0 order transmission increases for decreasing alignment period *Λ*.

## 4 Discussion

### 4.1 Three Different Metastable States

The good correspondence between simulated (Figures 3 and 4) and experimentally measured (Figure 1) POM images indicates that the origin of the 3 different electrically accessible metastable states has been correctly identified. The simulations provide insight in the director configuration and the underlying topology of the 3 different states. What discriminates state 1 from state 2 and 3 is the absence of singular disclination lines. The defect‐free director configuration is realized by inducing localized regions with a strong out‐of‐plane tilt of the director. The imposed anchoring conditions require the formation of a 2*Λ* × 2*Λ* unit cell with a bulk director configuration that covers the whole S^2^ unit sphere (including the north pole corresponding to vertical director orientation), in order to prevent defect formation.^[^ ^48^ ^]^ In state 2 and 3 on the other hand, the director remains predominantly planar (parallel to the substrates) in the bulk of the layer, which goes hand in hand with the presence of singular disclination lines. A (close to) uniform mid‐plane director orientation gives rise to the creation of a square grid of disclination lines (state 3), similar to what has been observed previously by Wang et al. in a cell geometry obtained by sliding substrate assembly.^[^ ^47^ ^]^ We here demonstrate that this state 3 is electrically accessible (by using switching path C, Figure 1i) in a cell with fixed geometry filled with DFLC. We also show that another configuration with diagonal disclination lines (state 2) can be reached when starting from the defect‐free configuration (state 1).

The director configuration of state 1 is well‐understood and described in full detail in previous articles, describing the alignment of nematic LC E7 (with positive dielectric anisotropy) in cells with the same type of surface anchoring pattern.^[^ ^48^, ^49^, ^50^, ^51^ ^]^ By filling the cells at high temperature and cooling down to room temperature, the defect‐free state 1 configuration has been obtained as the minimal free energy configuration in these previously studied examples. The tested (*Λ*, d) combinations were (*Λ*, d) = (6.5 µm, 3 µm); (3.2 µm, 3 µm) and (12.5, 10 µm), including devices with similar *Λ*/d ratios as investigated in this manuscript.^[^ ^48^, ^49^, ^50^, ^51^ ^]^ Although we here use DFLC material, providing additional options for post‐fabrication electrical switching, the origin of state 1 is the same as in the previously studied devices. The agreement between simulations (Figure 3) and experiments (Figure 1) for state 1 is excellent for both simulated alignment periods *Λ *= 4.5 µm and *Λ *= 9 µm. For the smallest alignment period of *Λ* = 2.6 µm the experimentally observed structure (Figure 1c) is deformed and is no longer recognized as a diagonal texture in POM. The same effect was observed previously and can be associated with the presence of unequal elastic constants as shown in Figure S14 (Supporting Information).^[^ ^49^, ^50^ ^]^ The smaller the alignment period and the larger the cell thickness (see Figures S6 and S11, Supporting Information), the more pronounced the effect of unequal elastic constants.

The distinct configuration in state 2 and 3, with another orientation of the disclination lines, is accompanied by a different behavior of the mid‐plane director. Different from state 3, the planar mid‐plane director in state 2 is periodically rotating along the diagonal direction (Figure 3). This rotation pattern gives rise to periodic splay‐bend energy modulations, while no elastic energy is associated with a uniform director orientation, as approximately seen in the mid‐plane of state 3. For decreasing alignment periods, the splay‐bend energy associated with the rotating director patterns increases, intuitively explaining why state 2 starts to deform (Figure 1c–e). The Q‐tensor simulations presented in Figure 4 indicate that this deformation is associated with a decrease in the rotation speed of the planar director observed in the middle of the cell (and therefore with a decrease in the elastic energy associated with this rotation). The collection of diagonal disclination lines is split up into 2 sets of disclination lines, closer to the top and bottom substrate respectively, that are making an angle 45° ± α with respect to the x‐axis. The smaller the alignment period, the larger the intersection angle 2α between the disclination lines becomes Figure 1c–e and the more the deformed state 2 starts to resemble a state 3 configuration, with a close to uniform mid‐plane director orientation and a 90° intersection angle between the disclination lines at the top and bottom. This is supported by the results in Figure 1, Figure S6, Figure S8 and Figure S11 (Supporting Information) that in general show increasing deviations from the non‐deformed state 2 for decreasing alignment periods *Λ*. Remark however that the periodicity of the deformed state 2 tends to break down over larger distances, with deviating angles α appearing at different positions in the cell (see Figure S3, Supporting Information). This hints that the formation of the deformed state 2 is sensitive to the presence of anchoring defects, dust particles, thickness uniformity, etc. For this reason the values for α in Figures 1 and Figure S6,S8, and S11 (Supporting Information) are only indicative and the comparison with the simulations in Figure 4, in which periodic boundary conditions are assumed, can only be made qualitatively. Overall the results in Figure 4b for a 8*Λ* × 8*Λ* periodic unit cell size and with α = 14°, better match the experimental results presented in Figure 1 (with α ≈ 12°, 14° and 17° for *Λ* = 6.5, 4.7, and 2.6 µm) than the results in Figure 4a with α = 27°. However, the match is not perfect and the simulations in Figure 4 are especially useful to understand the formation mechanisms of the deformed state 2, in which the rotation speed of the planar director is decreased in the middle of the cell.

Although switching paths B and C (Figure 1b) give rise to a different metastable end‐configuration in all tested geometries, the experiments indicate that the stability of deformed state 2 compared to state 3 is decreasing with decreasing alignment period *Λ* (indicating a higher free‐energy for the metastable state 2 configuration). This becomes especially apparent when looking at the results obtained in a cell with an alignment pattern with variable anchoring period as shown in Figure S5 (Supporting Information). Moreover, exerting a small pressure on a cell that is in the deformed state 2 (with small alignment period *Λ*), transforms the configuration into state 3 with a square grid of disclination lines. On the other hand, a large alignment period *Λ* increases the stability of state 2, making it more difficult to realize a uniform state 3 (see Figure S8d, Supporting Information).

The disclination lines that are present in state 3 are twist disclination lines, often referred to as reverse twist disclinations, because they separate regions with an inverse twist. M. Wang et al. have theoretically analyzed the forces acting on these kind of disclination lines, including the line tension, the interaction energy between disclination lines and the force coming from the patterned surface anchoring.^[^ ^47^ ^]^ The force balance determines the distribution and the stability of the disclination lines. The force induced by the periodically rotating surface anchoring patterns pulls the disclination lines with the same handedness down to the surface. The attractive force acting on the disclination lines is inversely proportional to the alignment period *Λ* (denoted as the pitch *p* in the manuscript of M. Wang et al.) and adds up with the repulsive force from the opposite substrate. This explains why the disclination lines in state 3 are more closely attracted toward the surface for smaller alignment periods *Λ*, as confirmed by our simulations (Figure 3e vs Figure 3f). The closer the disclination lines are to the surface, the larger the region with close to uniform director configuration in the bulk of the layer and the thinner the layers with a periodically rotating orientation. Remark that although an equal elastic constant approximation was used in the presented simulation results (Figure 3), the state 3 configuration is only weakly influenced by the exact values of the elastic constants as shown in Figure S14 (Supporting Information). It is the well‐chosen combination of orientationally patterned substrates (with a periodic rotation in perpendicular directions at both confining substrates), that enables the creation of a free‐standing grid of twist disclinations.

To conclude, it can be noted that some symmetry breaking is required to generate state 3 with one preferred mid‐plane director orientation. In Figure 1, a black region is observed in POM between crossed polarizers in the middle of the square pattern representing state 3. This hints that the mid‐plane director is either oriented along the analyzer or the polarizer direction, corresponding to the x‐ or y‐axis respectively in Figure 1. The simulation results presented in Figure 3e,f show a director configuration with a mid‐plane director that is roughly oriented along the x‐axis (with increasing modulations for smaller alignment period). However, an alternative solution with the mid‐plane director approximately along the y‐axis is shown in supporting information Figure S4 (Supporting Information). The disclination lines close to the top and bottom substrate in both cases are shifted over a distance *Λ*/2 along the y‐ and x‐axis respectively. This results in a rather similar appearance in POM, but subtle differences allow to distinguish both states. The bright lower‐left corner (rather than top‐right corner) that is experimentally observed in each *Λ* × *Λ* unit cell hints that the mid‐plane director orientation corresponds with the simulations presented in Figure 3. However, as shown in Video S7 (Supplementary Video), in our current experiment the defect grid sometimes moves during (or shortly after) the switching process until a stable position is maintained. To make the grid formation fully deterministic, symmetry breaking could be induced intentionally by introducing a predefined mid‐plane director orientation in state 3 (in the anchoring pattern or the electrode geometry).

### 4.2 Switching Paths Between the Different States

Successful identification of the director configuration and the defect topology (as discussed in section 4.1) allows to provide physical insight in the proposed switching mechanisms between the different states as presented in Figure 1g–i. To switch from state 1 to state 2 (path B, Figure 1h), the diagonal lines with vertical mid‐plane director orientation need to be destabilized and converted into a set of disclination lines. This is achieved by pulling the LC director toward a planar alignment with the help of an applied electric field in the negative dielectric anisotropy range (*f* > *f_co_*). The dielectric energy is strongly increased in regions with a vertical LC director orientation (roughly parallel to the electric field lines) and for sufficiently high electric fields the formation of state 2 is favored. The creation of disclination lines requires some energy but this is compensated by minimization of the dielectric energy due to the predominantly planar director orientation in state 2.

To switch from state 1 to state 3 on the other hand, the structural information that is contained in state 1 should first be erased with the help of a voltage application in the positive dielectric anisotropy range *f* < *f_co_* (path C, Figure 1i). In this way, the director becomes nearly vertically aligned everywhere in the bulk of the layer (with only minor importance of azimuthal rotations). By immediately switching from this state with vertical bulk alignment toward a state with planar bulk alignment, state 3 with a close to uniform mid‐plane director orientation is obtained. This last transition is controlled electrically, by adjusting the frequency of the applied electric field so that it becomes larger than the crossover frequency (*f* > *f_co,_* Δɛ < 0). As a result, the dielectric response favors a planar director orientation and the formation of a uniform mid‐plane director orientation is motivated by minimization of the elastic energy.

The switching mechanism from state 2 or state 3 to state 1 relies on the fact that state 1 is the minimum free energy configuration, containing diagonal line regions with vertical midplane director orientation but no singular disclinations. To reach this configuration, the disclination lines present in state 2 or 3 need to be destabilized and the structure subsequently needs to relax in a minimal free energy configuration in which localized regions with vertical director alignment are present. Voltage application with *f ≈ f_co_ is* very effective in destabilizing the disclination lines, motivating the first part of the switching path (Figure 1g), in which the frequency is varied from 5kHz (*f* > *f_co_*) to 1 kHz (*f* < *f_co_*). Remark that a variable frequency path was chosen to limit the sensitivity of the switching path to temperature changes: for moderate changes in temperature, the crossover frequency stays in the 1–5 kHz range, ensuring that the switching path remains effective. After obtaining an approximately defect‐free and mainly vertical aligned director configuration for an applied voltage with *f* < *f_co_* (*Δɛ* > 0), state 1 is obtained upon removal of the voltage.

**Figure 5:**
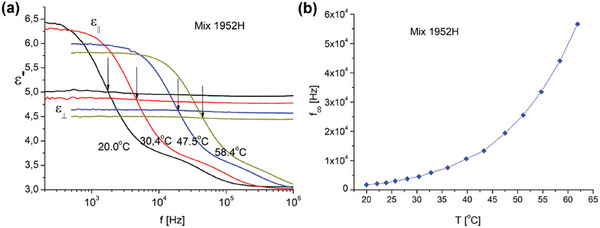
a) Dielectric properties of the LC mixture 1952H as a function of the frequency for 4 different temperatures between 20 and 58.4 °C. b) Cross‐over frequency *f*_*co*_for LC mixture 1952H as a function of the temperature. This figure is reused from Figure 2 in reference.^[^ ^41^ ^]^

Remark that although only one switching path is presented to reach the different states (respectively path A to reach state 1, path B to reach state 2 starting from state 1 and path C to reach state 3), a range of frequencies and applied voltages could be used to obtain the same end result. The switching mechanisms between the multi‐stable states make appropriate use of the positive or respectively negative dielectric anisotropy of the DFLC mixture to induce the required structural transformations, but there is some freedom to choose the applied frequency and voltage. To determine the frequency and voltage combinations presented in Figure 1g–i for DFLC 1952H and Figure S8e–g (Supporting Information) for DFLC 1999A, the following arguments have been considered:

- Switching paths requiring Δɛ > 0 can make use of different frequencies within the range *f* < *f_co_*. However the crossover frequency is temperature sensitive and just below *f_co_* the dielectric anisotropy is still relatively limited (**Figure** **5**a). The frequency is therefore chosen to be sufficiently lower than *f_co_* measured at room temperature, while still remaining high enough to avoid ionic effects. Frequencies in the range *f* = 100 Hz to 1 kHz have been tested and provide indistinguishable results (for the 1952H material).
- Switching paths B and C requiring Δɛ < 0 can make use of different frequencies within the range *f* > *f_co_*. However, the temperature sensitivity of *f_co_* makes it relevant to pick a frequency that is sufficiently higher than the crossover frequency measured at room temperature. On the other hand, it is preferable not to choose the frequency too high, to limit the dielectric heating (that scales roughly linearly with the frequency).^[^ ^53^ ^]^ These effects strongly depend on the material properties of the DFLC material, so the used material determines what can be a suitable frequency range. The 1952H material (as presented in the main manuscript) did not suffer from substantial dielectric heating, while considerable heating was observed for the tested switching paths for the 1999A material. Experimental testing confirmed that *f* = 30 kHz and *f* = 80 kHz for 1952H and 1999A respectively gives rise to reliable and repeatable switching results, in which the duration of the voltage application does not substantially affect the results (for the investigated voltage amplitudes). A higher frequency (*f* = 80 kHz) was chosen for the 1999A material to avoid temperature induced shifting of the crossover frequency (due to dielectric heating) above the applied frequency.
- After fixing the frequency values used to apply voltages in the positive and negative dielectric anisotropy range respectively, suitable amplitudes for the voltage were determined. The voltage was chosen high enough to achieve good switching between the different states, with a switching time that remains reasonably limited (10s of seconds). At the same time the voltage was kept relatively low to minimize energy consumption, to minimize the chances for dielectric breakdown and to minimize dielectric heating effects (that scale quadratically with the voltage).^[^ ^53^ ^]^ For the 1952H material, values of 8 V_rms_ for the Δɛ > 0 regime and 20 V_rms_ for the Δɛ < 0 regime were sufficient to reliably switch all the anchoring patterns (with different anchoring periods) in the 2 tested cells (with *d* = 3.5 µm and 4.5 µm). For the 1999A material, values of 8 V_rms_ for the Δɛ > 0 regime and 14 V_rms_ for the Δɛ < 0 regime were sufficient for all anchoring periods and for both cells (with *d *= 3.3 µm and 5.4 µm). The minimal required voltages were somewhat higher in the thicker cells, but for uniformity we used the same voltages in the tested cells with slightly different thickness.

### 4.3 Diffraction Characteristics

The experimentally measured diffraction properties are rather well reproduced by the simulation results, confirming both the symmetry of the diffraction pattern and providing a reasonably good agreement for the measured diffraction efficiencies in the 0 order (as shown in Figure 2a). Although infinitely strong anchoring conditions and an equal elastic constant approximation was used to determine the director distribution, a rather good qualitative agreement is obtained.

When looking at the symmetry of the measured and simulated diffraction patterns, some questions could be raised comparing the diffraction pattern in state 1 (Figure 2d,f) with the simulations in Figure 3a,b. Visual inspection of the diffraction patterns in state 1 (Figure 2d,f) shows an almost equal intensity in all 4 (+− ½, +− ½) orders, while some asymmetry is observed in the simulations (Figure 3a,b), with the (½,½) and (−½,−½) orders being more prominent. Comparison with Figure S7 (Supporting Information) (showing the experimental diffraction pattern for a somewhat thicker cell with *d* = 4.5 µm), demonstrates that the apparent symmetry observed in (Figure 2d,f) is not universally linked to the director configuration in state 1, but is induced by a specific combination of different parameters (cell thickness, birefringence, etc.). The slight discrepancy with the simulations in Figure 3a,b can therefore be linked to several inaccuracies or assumptions: non‐uniformity in the cell thickness, unknown material birefringence at 633nm (with the value at 589 nm being used in simulations), the equal elastic constant assumption and the infinitely strong anchoring assumption in the simulations.

When comparing the values for the 0 order transmission, only the simulated 0 order transmission for the deformed state 2 with *Λ* = 4.5 µm substantially deviates from the measured value for *Λ* = 4.7 µm. The simulated result is sensitive to the exact height of the disclination lines in the LC layer (being influenced by the LC elastic constants) and does not take into account that the structure is not perfectly periodic over larger distances. As shown in Figure S3 (Supporting Information), the periodicity of the deformed state 2 tends to break down over large areas, with deviations in the orientation angle of the disclination lines showing up over larger distances. This could give rise to an increased 0 order transmission, as experimentally observed (Figure 2a).

The exact properties of the diffraction pattern and the intensity distribution strongly depend on the geometrical parameters (cell thickness, alignment period) and material parameters (especially birefringence), but it is clear that the proposed device allows switching between 3 distinct optical states that are all metastable without applied voltage. For the presented device, with a thickness *d* = 3.5 µm and filled with DFLC 1952H with Δ*n* = 0.101, the state 3 configuration shows the largest 0 order transmission while state 2 is diffracting most of the light and state 1 has an intermediate behavior. The transparency of state 3 can be strongly increased by decreasing the surface alignment period, bringing the disclination lines closer to the surfaces and in this way increasing the contribution from the uniformly aligned director configuration (not inducing any diffraction) in the bulk of the cell (Figures 2 and 3). For a sufficiently small alignment period, the surface layers become so thin that the induced retardation and the accompanying diffraction is almost negligible, leading to a highly transparent state that could be interesting for smart window applications. Similar conclusions hold when using another LC material or another cell thickness (see supporting information Figure S13, Supporting Information). The diffraction behavior of state 1 and (deformed) state 2 is more difficult to understand intuitively and simulations are required to describe the behavior. Although state 2 has the lowest 0 order transmission in the presented cell (*d* = 3.5 µm, Δ*n* = 0.101), experiments with another cell thickness or another DFLC material (as presented in Figures S7,S10,S12 and S13 in the Supporting Information) have shown that state 1 can also become the least transparent, with state 2 showing some intermediate behavior. This strongly depends on the cell thickness and material birefringence, leaving room for optimization of the diffraction properties depending on the envisioned application (smart windows, beam steering, etc.). Remark however that the proposed switching mechanism between the 3 multi‐stable states will eventually break down for certain parameter combinations. The reported results demonstrate that multi‐stability between 3 different states can be obtained for all tested alignment periods and cell thicknesses (with the following ranges being investigated *Λ* = 2.6–15 µm and *d* = 3.3 µm–5.4 µm). However, the results should be reevaluated for strongly deviating geometrical parameters, for which the multi‐stability of the same 3 states is not a priori guaranteed.

Even though the focus in this work is on the stabilization of different metastable states without the need for an externally applied voltage, it can be foreseen that the detailed properties of the individual states can still be modified with the help of a (continuous) voltage application. Starting from a stable configuration, applying a moderate voltage that does not induce a transition toward another state, will modify the director configuration and optical properties. As demonstrated before, starting from state 1 a voltage in the frequency range with positive dielectric anisotropy can be applied to tune the diffraction properties.^[^ ^49^, ^50^ ^]^ However, a limited tuning of the properties in state 2 and 3 could be foreseen as well and the use of different applied frequencies (below and above the cross‐over frequency) in a DFLC mixture further expands the possibilities. A detailed investigation is preserved for future studies.

Finally, also note that the versatility of the photoalignment technique allows to easily adjust the cell design, for example introducing a variable alignment period or a smaller rotation angle between the top and bottom substrate.^[^ ^54^, ^55^ ^]^ Variations of the rotation angle between the top and bottom substrate have been outside the scope of this work, but X. Wang et al. have recently studied these so‐called “Moire patterns” with a small rotation angle (<50°) between the top and bottom substrates by manually rotating the substrates with respect to each other.^[^ ^46^ ^]^ Depending on the ratio between cell thickness and alignment period, several textures have been observed for a fixed rotation angle. Electric field induced switching between some of these textures could potentially be achievable by using similar methods as the ones described in this manuscript. Even though one texture with minimal free energy exists, configurations with a somewhat higher free energy might be electrically accessible in a DFLC mixture and could remain metastable after removal of the voltage.

## 5 Conclusion

Experimental measurements have demonstrated that 3 different multi‐stable bulk LC configurations can be achieved in cells with crossed assembly of periodically rotating anchoring patterns at the top and bottom substrate. These 3 different bulk configurations are electrically accessible by using a dual‐frequency LC material and remain metastable after removal of the voltage. One configuration is defect‐free, while the 2 other configurations contain a network of disclination lines with a different topology. The frustration induced by the crossed assembly of 2 rotating anchoring patterns is resolved in the defect‐free configuration by introducing regions with a vertical mid‐plane director orientation. The planar bulk director orientation in the other 2 states leads to the creation of twist disclination lines. The frequency‐dependent dielectric properties of the DFLC are crucial to enable reliable and repeatable switching between the different states. The regions with vertical mid‐plane alignment are destabilized by applying a frequency in the negative dielectric anisotropy range, leading to the creation of disclination lines. The topology of the disclination network however depends on the switching path, that can either include an antecedent voltage application in the positive dielectric anisotropy range (to reach state 3) or not (to reach state 2). After electrically driving the LC into a defect‐containing configuration, the obtained configuration becomes metastable, retaining its topological features after removal of the voltage. Disclination lines are entities with a remarkable stability and the topological energy barrier that is present between the different states avoids the relaxation in the minimal free energy configuration. From an application‐oriented perspective, the observed metastability without the need for an applied voltage offers opportunities for low‐power devices that only require power to switch from one state to another. The presented results demonstrate that very distinct optical properties can be associated with each metastable state and that these properties are strongly affected by the geometrical parameters (cell thickness, alignment period) and the material properties (birefringence, elastic constants). This allows to carefully engineer the device design depending on the envisioned application, for example in smart windows or beam steering devices. In general, the robust control over disclination lines in LCs is deemed to enable novel devices with tailored properties and the demonstrated electrical driving offers a technologically relevant control mechanism. Apart from optically driven applications, the reliable switching between different disclination networks and/or defect free configurations in LCs could drive further research into colloidal assembly, micro‐transport and stimuli‐responsive actuators.

## 6 Experimental Section

### Cell Fabrication

The ITO coated glass substrates (Delta Technologies) are first thoroughly cleaned and then placed in an UV‐ozone chamber (15 min with UV illumination at 90 °C) to improve the quality of the subsequent coating step. After cooling the substrates down to room temperature, a solution of 0.5 wt.% SD1 in DMF is spin‐coated onto the ITO‐side of the substrates at 3000 rpm for 30 s. After spinning, the substrates are soft‐baked for 5 min at 90° to evaporate the solvent.

To create the photoalignment patterns, the coated substrate (further named sample) is placed in an interference setup making use of a blue laser (Cobolt Twist, λ = 457 nm) that emits linearly polarized light. A combination of a half wave plate and a polarizing beam splitter is used to split the beam into 2 orthogonally polarized beams with equal intensities. One of the beams is redirected toward the sample via a mirror, so that both beams interfere in the sample plane, making the same angle with respect to the substrate normal. Both beams propagate through a quarter wave plate before reaching the sample, which transforms them into a left‐ and right‐handed circularly polarized beam respectively. At the sample, the 2 circularly polarized beams with opposite handedness create an interference pattern with a close to linear polarization pattern that is periodically rotating in space.^[^ ^56^ ^]^ The half‐opening angle β between both beams determines the photoalignment period *Λ* over which the director rotates by 180°, according to *Λ* = 457 nm/(2 sin(β)). Anchoring patterns with different alignment periods (*Λ* = 2.6, 4.7, 6.5, and 9.1 µm) are written at different areas on the same substrate and 2 substrates are separately illuminated to allow fabrication of a cell afterward. The anchoring patterns are inscribed by using a 100 mW illumination power during 40 s and the diameter of the illuminated spots was ≈8 mm.

Before cell assembly, one of the photo‐aligned substrates is rotated over 90°, in order to produce the anchoring pattern displayed in Figure 1a. The positioning of the areas with different photo‐alignment periods (*Λ* = 2.6, 4.7, 6.5, and 9.1 µm) is controlled, in order to obtain the desired anchoring pattern (with the same alignment period at the top and bottom substrate) in the produced cell. Since the anchoring patterns on both substrates are 1D periodic (without variation in the 2nd dimension), no high‐precision alignment of the substrates is required. The overlap between the photopatterned areas at both substrates needs be ensured, requiring an accuracy for the cell assembly that is related to the dimension of the illuminated areas (and not to the anchoring period *Λ)*. This makes manual assembly feasible for the cells presented here (with spot diameter ≈ 8 mm), but additional control mechanisms are required when the illuminated areas are very small or the rotation angle between both substrates needs to be controlled very accurately. As described in the supporting information, the deposition and polymerization of a thin reactive mesogen (RM) layer on top of the photopatterned substrates, allows for a more precise alignment of the illuminated areas at both substrates on top of each other. The thin RM layers provide a small retardation, making the alignment patterns visible in POM and allowing to control the relative positioning of the alignment patterns before curing the glue (and in this way fixing the cell assembly). The substrates are glued together with UV curable NOA68 glue containing spherical spacer balls with a thickness of ≈ 3µm. After soldering 2 copper wires, the cell is filled with DFLC material at elevated temperature (90 °C) and cooled down to room temperature.

### DFNLC Material Properties

The material properties of the DFLC 1952H, prepared at Warsaw Military University (Poland) and being the modification of mixtures AB1 previously reported by Dabrowski et al.,^[^ ^57^ ^]^ are listed in **Table** **1**. The nematic to isotropic transition temperature for the 1952H mixture is 154.8 °C, while crystallization only occurs below 0 °C. The dielectric properties of the LC mixture 1952H as a function of the frequency are shown in Figure 5a for 4 different temperatures between 20 and 58.4 °C. The temperature dependence of the cross‐over frequency f_co_ is shown in Figure 5b.

**Table 1:** Optical and dielectric properties of DFLC material mixture 1952H at 20 °C.

Optical properties	Dielectric properties		
Δ*n* (at 589 nm)	0.101	Δɛ (at 1 kHz)	1.37
*n*_o_	1.4757	Δɛ (at 1 MHz)	−2.10
*n*_e_	1.5765	*f*_*co*_	1.74 kHz

### FE Q‐Tensor Simulations

Finite element Q‐tensor simulations, initially developed at University College London, are used to find different metastable solutions for the LC configuration in the photopatterned cells.^[^ ^58^, ^59^ ^]^ The LC is represented with the help of a second order tensor Q and solutions with a local minimum in the free energy are found by minimizing the Landau‐de Gennes free energy functional starting from a well‐chosen initial condition. The total free energy, being the sum of elastic distortion energy, thermotropic (or Landau) energy, electrical energy and surface energy, can be expressed as a function of the Q tensor and its spatial derivatives. Strong anchoring conditions are assumed at the top and bottom substrate, meaning that the anchoring cannot deviate from the planar anchoring pattern that is imposed by the photoalignment pattern. This pattern is described by a 0° pretilt angle (with respect to the xy‐plane) and an azimuthal anchoring angle φ (with respect to the x‐axis) equal to φ = π/2–π x/*Λ* at the bottom substrate and φ = π/2–π y/*Λ* at the top substrate. Periodic boundary conditions are used along the x‐ and y‐direction, simulating a 2*Λ* × *2Λ* periodic unit cell for the simulations presented in Figure 3 and a 4*Λ* × 4*Λ* or respectively 8*Λ* × 8*Λ* periodic unit cell in Figure 4a,b. The simulated cell thickness is *d* = 3.5 µm and 2 different alignment periods of *Λ* = 4.5 and 9 µm are simulated. An equal elastic constant approximation is used with K = 12 pN (see Figure S14 (Supporting Information) for the use of other elastic constants). The bulk thermotropic coefficients are based on the ones measured for 5CB at a reduced temperature of −2 °C (A = −174 N m^−2^, B = −2120 N m^−2^, C = 1740 N m^−2^), giving rise to an equilibrium order parameter of 0.54.^[^ ^60^ ^]^ The disclination lines in state 2 and 3 are indicated in Figures 3 and 4 by plotting the regions with a reduced order parameter equal to 0.35. A sufficiently small tetrahedral mesh is used to ensure the mobility of the disclination lines during the optimization process.

### Optical Simulations: POM Images and Far‐Field Diffraction Characteristics

The simulated POM images are generated with an open‐source optical simulation package in Python, called Nemaktis (https://github.com/warthan07/Nemaktis).^[^ ^61^ ^]^ Within this tool, the acquired director vector field from the FE‐element simulations is given as an input to describe the LC layer. A generalized beam propagation method is used to calculate the electromagnetic fields through the LC cell, recasting Maxwell equations for birefringent media in a simpler Schrödinger‐like propagation equation for the transverse field.^[^ ^61^ ^]^ This method separates the cell into multiple slabs, each with a z‐independent permittivity tensor and stitches the results of the separate slabs back to each other.^[^ ^61^ ^]^ The propagation equation contains a contribution to the phase evolution (similar to the Jones method), a diffraction operator and a walk‐off‐operator. Calculations are performed for x‐ and y‐polarized light as input. A GUI is used to visualize the results. The measured refractive indices at 𝜆 = 589 nm (*n*_o_ = 1.4757, *n*_e_ = 1.5765) for the LC mixture 1952H are taken into account in the optical simulations and 15 equally spaced discrete wavelengths between 450 and 750 nm are used to create color images, making use of the spectrum of the CIE illuminant A. The focusing optics in the microscope are accounted for by using the well‐known formula of Fourier optics, with a numerical aperture of 0.3 for the objective taken into account to generate the simulated POM images in Figure 3, Figure 4, Figure S4 and S14 (Supporting Information).

The diffraction patterns are generated by first calculating the near‐field transmission pattern for the same wavelength as the laser in the experimental setup (λ = 633 nm) with Nemaktis. The near‐field components are transposed to the far field by using a 2D spatial Fourier transform following the Fresnel‐Kirchoff formulation. This was done by using a 2D discrete fast Fourier transform available in the numpy library in Python. The resulting intensities of each discrete order are normalized over the total intensity of all available orders.

### Measurement Setups

The POM images were captured with the help of a Nikon eclipse Ci POL microscope, using a CFI P Achromat 20X objective. The angle for the polarizer and analyzer can be chosen independently. The camera itself is the UI‐1460SE‐C_HQ, a CMOS Color camera with 2048×1536 Pixel resolution. In the presented results, the analyzer and the polarizer are crossed, aligned along the x‐ and y‐axis respectively. In the supporting information (Figure S3, Supporting Information) images without polarizer and analyzer are shown as well.

The diffraction properties of the constructed 2D LC gratings were measured by making use a helium‐neon laser (*𝜆 *= 633 nm, JDS Uniphase). Linearly polarized light (with a beam diameter of ≈1 mm) was incident perpendicularly to the sample and the diffracted power in transmission was measured with the help of a photodetector (Newport 918D – SL‐ 0D3R) attached to a power meter (Newport 2936‐C). Each grating (with alignment period *Λ* = 2.6, 4.7, 6.5, or 9.1 µm) is illuminated separately and the diffraction pattern is recorded on a black screen that is placed behind the sample. Images are captured with the help of a cellphone camera. The diffraction efficiency is defined as the measured power in the diffraction spot divided by the measured power of the incident laser beam in front of the sample. The assigned labels in the (k_x_, k_y_) diagram are based on the diffraction equation for perpendicularly incident light *n*_out_ sin(θ_out_) = mλ/*Λ*, with m the diffraction order*. A* (p,q) label in the (k_x_, k_y_) diagram is associated with the pth diffraction order along the k_x_ direction (associated with a spatial period *Λ*/p in the x‐direction) and the qth diffraction order along the k_y_ direction (associated with a spatial period *Λ*/q in the y‐direction). According to this definition, non‐integer diffraction orders originate when the spatial period of the LC configuration is a multiple of the surface alignment period *Λ*.

## Conflict of Interest

The authors declare no conflict of interest.

## Supporting information



Supporting Information

Supplementary Video

Supplementary Video

Supplementary Video

Supplementary Video

Supplementary Video

Supplementary Video

Supplementary Video

Supplementary Video

Supplementary Video

Supplementary Video

Supplementary Video

Supplementary Video

## Data Availability

The data that support the findings of this study are available from the corresponding author upon reasonable request.
