# Supplementary material for: Electrically Switchable Multi‐Stable Topological States Enabled by Surface‐Induced Frustration in Nematic Liquid Crystal Cells
Source: Adv Mater. 2025 Jan 10;37(12):2414675. doi: 10.1002/adma.202414675 (PMC11938017; doi:10.1002/adma.202414675)
Supplement: Supplementary file 1 — Supporting Information [file ADMA-37-2414675-s013.pdf]

ADVANCED MATERIALS

Supporting Information

for *Adv. Mater.*, DOI 10.1002/adma.202414675

Electrically Switchable Multi-Stable Topological States Enabled by Surface-Induced Frustration in Nematic Liquid Crystal Cells

*Jelto Neirynck, Yu-Tung Hsiao, Migle Stebryte and Inge Nys**

Supporting Information

Electrically switchable multi-stable topological states enabled by surface induced frustration in nematic liquid crystal cells

*Jelto Neirynck, Yu-Tung Hsiao, Migle Stebryte, and Inge Nys**

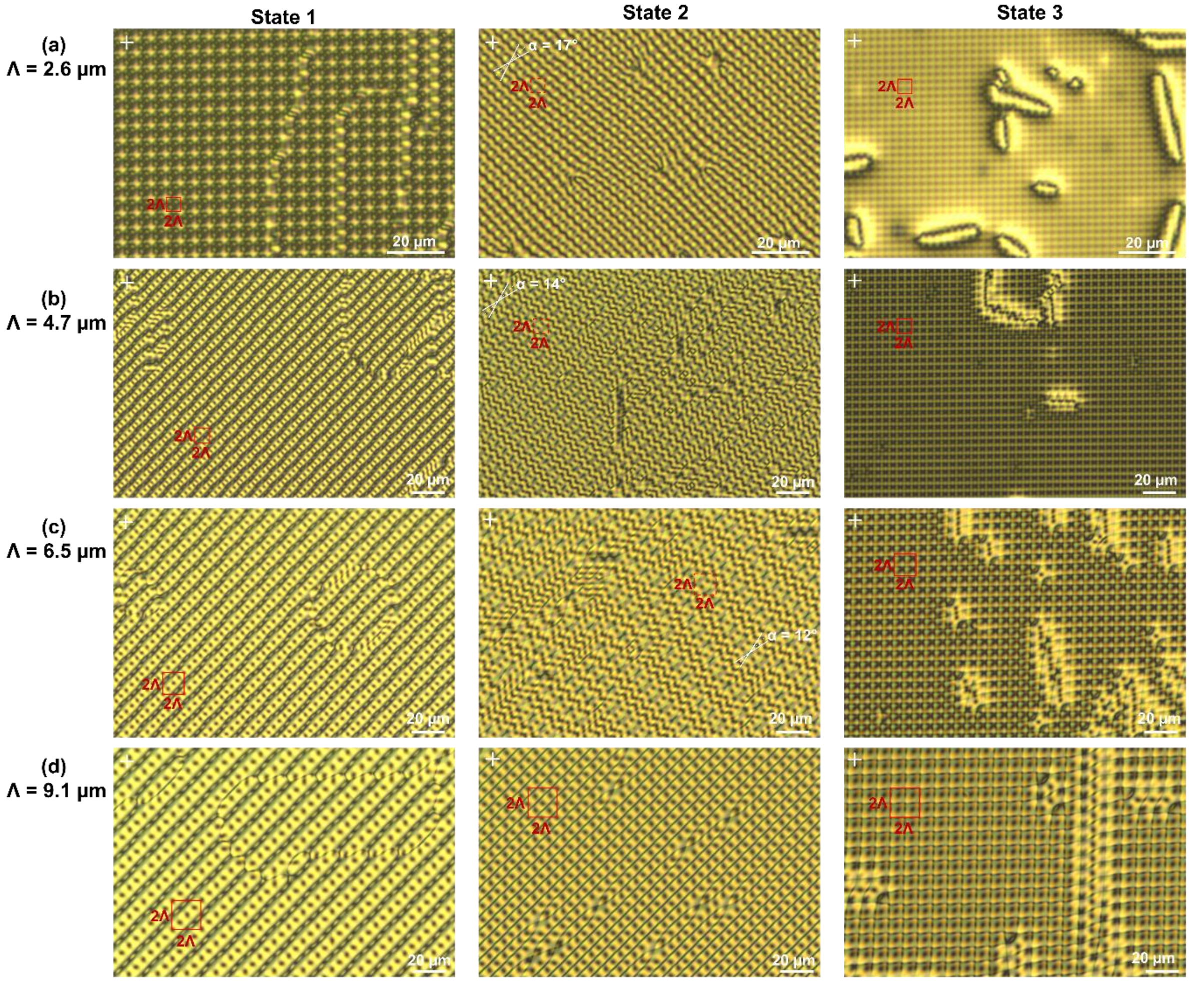

Figure S1: Polarizing optical microscopy images of state 1 (left), state 2 (middle) and state 3 (right) between crossed polarizers for alignment period Λ = 2.6 μm (a), Λ = 4.7μm (b), Λ = 6.5 μm (c) and Λ = 9.1 μm (d). The cell thickness is d = 3.5 μm and the used DFLC material is 1952H. Images are shown for a larger area compared to what is shown in Figure 1, showing some imperfections that appear over larger distances. In state 1, some domains can be seen that are shifted over a distance Λ in the x- and/or y-direction. Four equivalent positions exist in the 2Λ x 2Λ unit cell, allowing for the appearance of shifted domains that are separated by a

disclination line. [1-3] In state 2 and state 3 some imperfections can be observed as well, in which disclination lines are shifted to an alternative equilibrium position defined by the periodic photoalignment pattern.

Switching path A: DFLC 1952H

Different paths were tested to optimise the uniformity of state 1 after switching. A few of these paths are visualised in supporting **Figure S2**. It was observed that simply applying a frequency in the positive dielectric regime is insufficient. This was the case for the regular voltage (8 V_{rms}, Figure S2(a) and supporting **Video S1**) and for a higher value (20 V_{rms}, Figure S2(b) and supporting **Video S2**). Crossing f_{co} once helps to destabilise the director configuration, which results in a more uniform state 1 (Figure S2(c) and supporting **Video S3**), but decreasing the voltage steadily or crossing the f_{co} twice (Figure S2(d) and supporting **Video S4** and Figure S2(e) and supporting **Video S5**) did not substantially improve the uniformity of the state 1 configuration further. Remark that the videos are shown for the d = 4.5 µm thick cell produced with the SLM setup (as discussed below). The quality of these samples was somewhat lower than the sample reported in the main manuscript, explaining the comparatively large amount of observed defects compared to Figure S1 (d = 3.5 µm, 1952H, produced with the interference photoalignment setup and without polymerized RM layers).

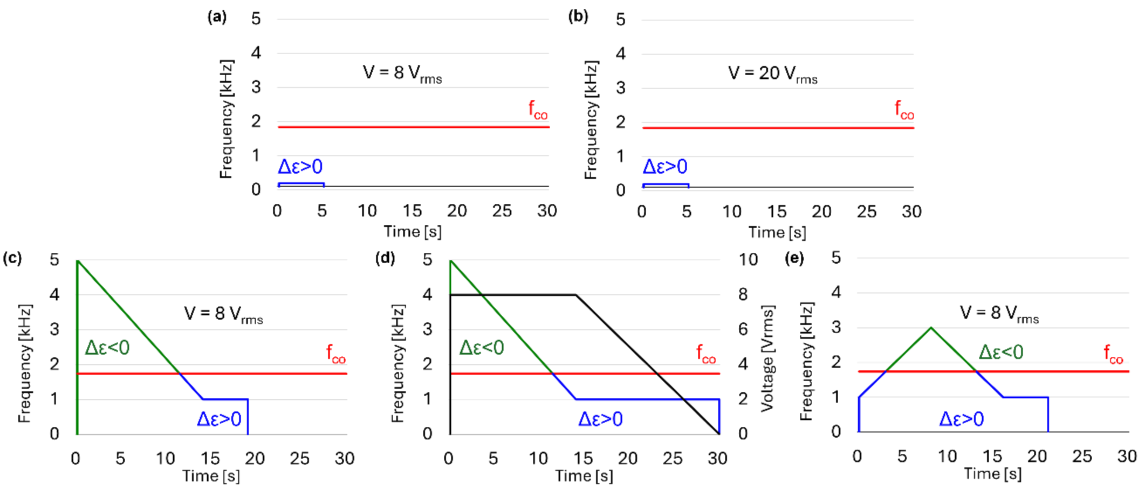

Figure S2. Different examined switching paths A for DFLC 1952H; (a) Applying a constant frequency of 100 Hz at 8 V_{rms} for 5 s, (b) Applying a constant frequency of 100 Hz at 20 V_{rms} for 5 s, (c) Gradually decreasing the frequency and crossing the f_{co} from 5 kHz to 100 Hz at 8 V_{rms} and maintaining the voltage for 5 seconds, (d) Gradually decreasing the frequency and crossing f_{co} from 5 kHz to 100 Hz at 8 V_{rms} and reducing the voltage steadily to 0 V_{rms},, (e) Crossing f_{co} twice by starting at 100 Hz, increasing the frequency to 3 kHz and reducing it back

to 1 kHz. The voltage is then directly turned off. The corresponding Videos are shown in S1-S5.

Supplementary Video S1: this video shows the switching from state 3 to state 1 by applying a constant frequency of 100 Hz and a voltage of 8 Vrms for 5 seconds. This method gives rise to a mixed state 1 and state 3 configuration and is sensitive to the amplitude of the applied voltage (see Video S2). Moreover, the method becomes completely inappropriate for the 1999A material, as shown in Video S8 and S9. The cell thickness is d = 4.5 µm, the DFLC material is 1952H and the video was made for alignment period Λ = 10 µm. The path is visualized in Figure S2(a).

Supplementary Video S2: this video shows the switching from state 3 to state 1 by applying a constant frequency of 100 Hz and a voltage of 20 Vrms for 5 seconds. This method is insufficient to reach a uniform state 1 (but the structure relaxes to state 3). The cell thickness is d = 4.5 µm, the DFLC material is 1952H and the video was made for alignment period Λ = 10 µm. The path is visualized in Figure S2(b).

Supplementary Video S3: this video shows the switching from state 3 to state 1 by gradually reducing the frequency from 5 kHz to 100 Hz at a constant voltage of 8 V_{rms} and maintaining this voltage for 5 seconds after reducing the frequency. This path is the same as the path A in Figure 1(g) and works to achieve a relatively uniform state 1. The cell thickness is d = 4.5 µm, the DFLC material is 1952H and the video was made for alignment period Λ = 10 µm. The path is visualized in Figure S2(c).

Supplementary Video S4: this video shows the switching from state 3 to state 1 by gradually reducing the frequency from 5 kHz to 100 Hz and gradually reducing the voltage afterwards. This path works to achieve a relatively uniform state 1, but does not improve further on the path shown in Figure S2(c) as only a statistically irrelevant deviating number of defects/domains is achieved. The cell thickness is d = 4.5 µm, the DFLC material is 1952H and the video was made for alignment period Λ = 10 µm. The path is visualized in Figure S2(d).

Supplementary Video S5: this video shows the switching from state 3 to state 1 by crossing f_{co} twice by starting at 100 Hz, increasing the frequency gradually to 3kHz and reducing the frequency back to 100 Hz again while maintaining a constant voltage of 8 V_{rms}. This path works

to achieve a relatively uniform state 1, but does not improve further on the path shown in Figure S2(c). The cell thickness is d = 4.5 µm, the DFLC material is 1952H and the video was made for alignment period Λ = 10 µm. The path is visualized in Figure S2(e).

Supplementary Video S6: this video shows the switching from state 1 to state 2, following switching path B in Figure 1(h). The cell thickness is d = 3.5 µm, the DFLC material is 1952H and POM images captured for alignment period Λ = 9.1 µm are shown.

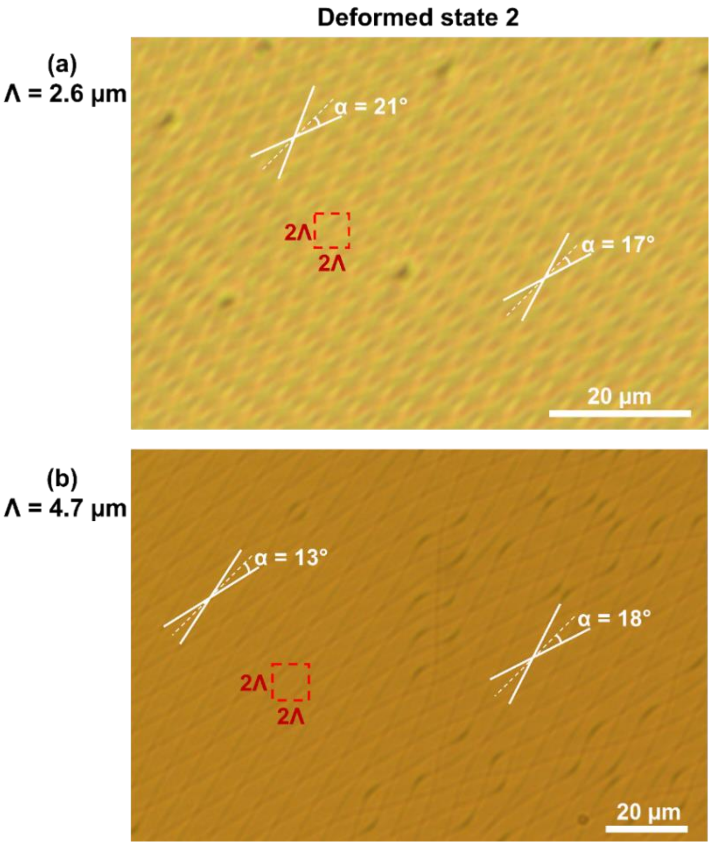

Figure S3: POM images without polarizers for deformed state 2, with indication of the orientation angle 45° + α and 45° - α of the two sets of disclination lines (closer to the top and bottom substrate). The cell thickness is d = 3.5 µm, the DFLC material is 1952H and results are shown for alignment period Λ = 2.6 µm (a) and Λ = 4.7µm (b). The smaller the surface alignment period, the larger the value for α becomes, leading to a larger intersection angle of the disclination. The periodicity of this deformed state 2 structure tends to break down over large areas, with deviations in the orientation angle of the disclination lines showing up over larger distances. Although the disclination lines are hardly visible in POM between crossed polarizers for these small alignment periods Λ (Figure 1(c,d)), POM images without polarizers more clearly reveal the presence of disclination lines.

Supplementary Video S7: the video shows the unstable position of the defect grid in state 3 during switching while the external field remains on. The defect grid moves during (or very shortly after) the switching process until a stable position is maintained.

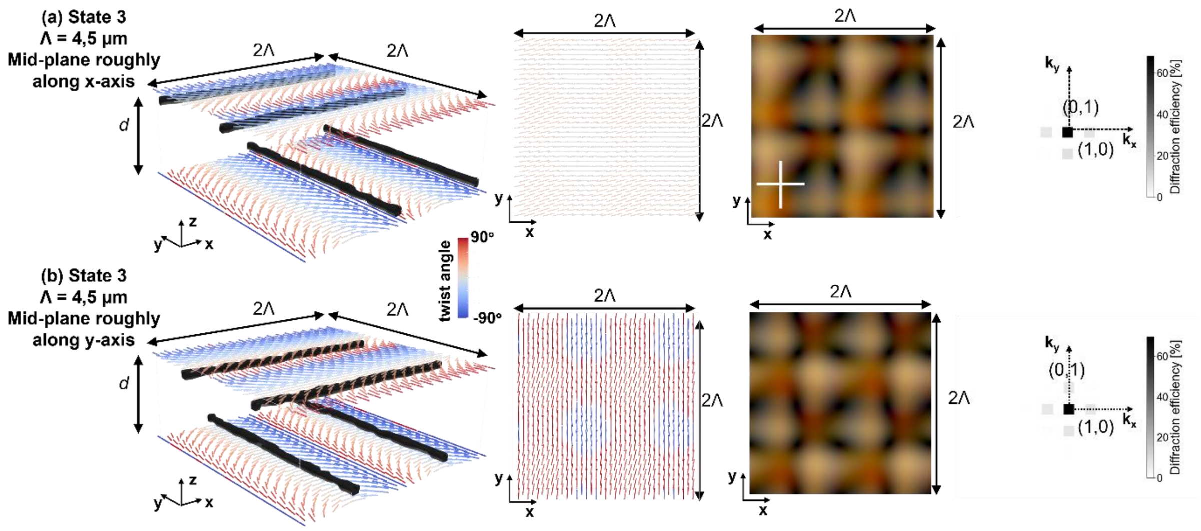

Figure S4. Simulated metastable director configurations without applied voltage in a cell with thickness d = 3.5 μm and alignment period Λ = 4.5 μm, assuming equal elastic constants K = 12 pN. Two possible results for state 3 are shown, with a planar mid-plane director orientation roughly along the x- or y-axis respectively in (a) and (b). From left to right the following simulation results are shown: a 3D view, the mid-plane cross-section for the director orientation, the simulated POM image and the simulated diffraction pattern for red light λ = 633 nm. The color bar represents the twist angle with respect to the x-axis. Regions with a reduced order parameter (equal to 0.35) are shown in grey, representing the disclination lines. When comparing (a) and (b), the disclination lines close to the top and bottom substrate are shifted over a distance $\Lambda/2$ along the y- and x-axis respectively.

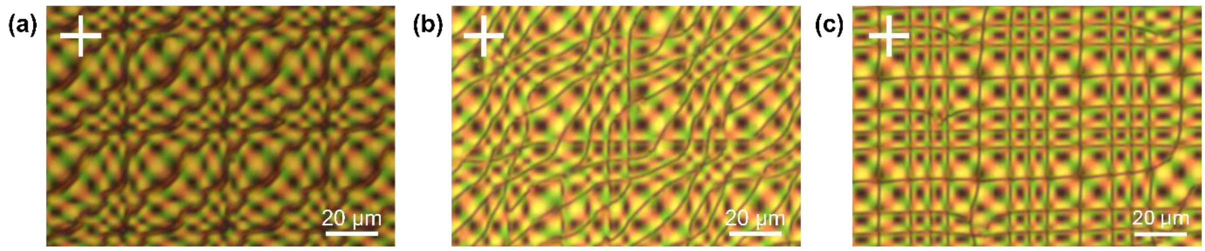

Figure S5. State 1 (a), state 2 (b) and state 3 (c) for a cell of 3.3 μm thickness with variable alignment period in which the mesogens follow the formula $\phi(x) = \frac{\pi x}{\Lambda} + \alpha\beta \sin\left(\frac{\pi x}{\beta\Lambda}\right)$ with Λ = 10 μm, α = 0.4 and β = 2 filled with DFLC material 1999A.

Cell fabrication with SLM setup

A different illumination method was used for the 4.5 µm thick cell filled with DFLC 1952H and the 3.3 µm and 5.4 µm thick cell filled with DFLC 1999A. An SLM-based illumination setup was used for the photoalignment patterning in these cells, which allows for more freedom to create unique alignment patterns (deviating from a pure periodic rotation).[4-7] The SLM setup consist of the same blue Cobolt Twist laser (λ = 457 nm) as in the interference setup. The light travels through the first quarter waveplate (QWP), which transforms the vertically polarized light into circularly polarized light. This light then reflects from the SLM, which takes a greyscale image to determine the needed voltage and tilt angle of the LC mesogens of each pixel in the parallel aligned nematic (PAN) LC panel. After reflecting, the light consists of a wavefront of different elliptically polarized light which differ per pixel, and travels through a second QWP which transforms it back into a wavefront of different linear polarized light.[2] The greyscale pattern imposed on the pixels of the SLM determines the local orientation of the linearly polarized light incident on the sample, which in turn determines the orientation of the photoactive molecules of the SD1 layer (perpendicular to the polarization).

Before cell assembly, an 4 wt% RM257 and 0.5-1 wt% Irgacure651 (IRG651) and 0.01 wt% tert-Butyl acrylate (tert-B) dissolved in toluene is spin coated on top of the photoactive SD1 layer at 2000 rpm for 30 s. Afterwards, the samples are placed under vacuum on a heating stage at 85°C. A UV lamp is placed over the heating stage and the samples are illuminated for 5 minutes. This process results in a thin RM layer which allows us to align the substrates optically under a POM by hand. This is needed to prevent misalignment between the anchoring patterns at the top and bottom substrate since the illuminated areas are only 1.53x0.86 mm^2 large (for the samples reported in the supporting information). After alignment of the top and bottom substrate under the POM, the substrates are glued together with UV curable NOA68 glue containing spherical spacer balls. After soldering two copper wires, the cell is filled with DFLC material at elevated temperature (90°C) and cooled down to room temperature.

Material properties: DFLC 1999A

Table S1. Material properties of DFLC mixture 1999A at 25°C

Optical properties		Dielectric properties	
Δn (at 589 nm)	0.2955	Δε (at 1 kHz)	3.70
n_o	1.5047	Δε (at 1 MHz)	-3.00
n_e	1.7972	f_{co} (kHz)	10.0

Experimental results: different DFLC materials and different cell thicknesses

In the main manuscript, detailed results are reported for the DFLC material mixture 1952H (received from Warsaw Military University, Poland) and a cell thickness d = 3.5 µm. **Figure S6** and **Figure S7** show the experimental results for a d = 4.5 µm cell filled with DFLC mixture 1952H. **Figure S8, Figure S10, Figure S11** and **Figure S12** also show experimental results for cell filled with DFLC mixture 1999A (received from Warsaw Military University, Poland, material properties see **Table S1**). The nematic to isotropic transition temperature for the 1999A mixture is 141°C, while crystallization typically only occurs below 0°C. The results for the measured zero order diffraction efficiency are provided in **Figure S13**. For material mixture 1999A (with very different viscoelastic and dielectric properties, Table S1 and ref.[8]), the switching voltages and frequencies have to be adapted (Figure S8(e-g)) but the same three different fundamental states are retrieved (in the two cells with different thicknesses d = 3.3 µm and d = 5.4µm). For the device presented in the main manuscript, with thickness d = 3.5 µm and filled with DFLC 1952H with Δn = 0.101, the state 3 configuration has the largest 0 order transmission while state 2 is diffracting most of the light and state 1 has an intermediate behavior (Figure 2). However, this behavior strongly depends on the cell thickness and material birefringence, leaving room for optimization of the diffraction properties depending on the envisioned application. This is demonstrated by the results in Figure S13. In these configurations, with another cell thickness or another DFLC material state 1 is the least transparent, with state 2 shows some intermediate behavior.

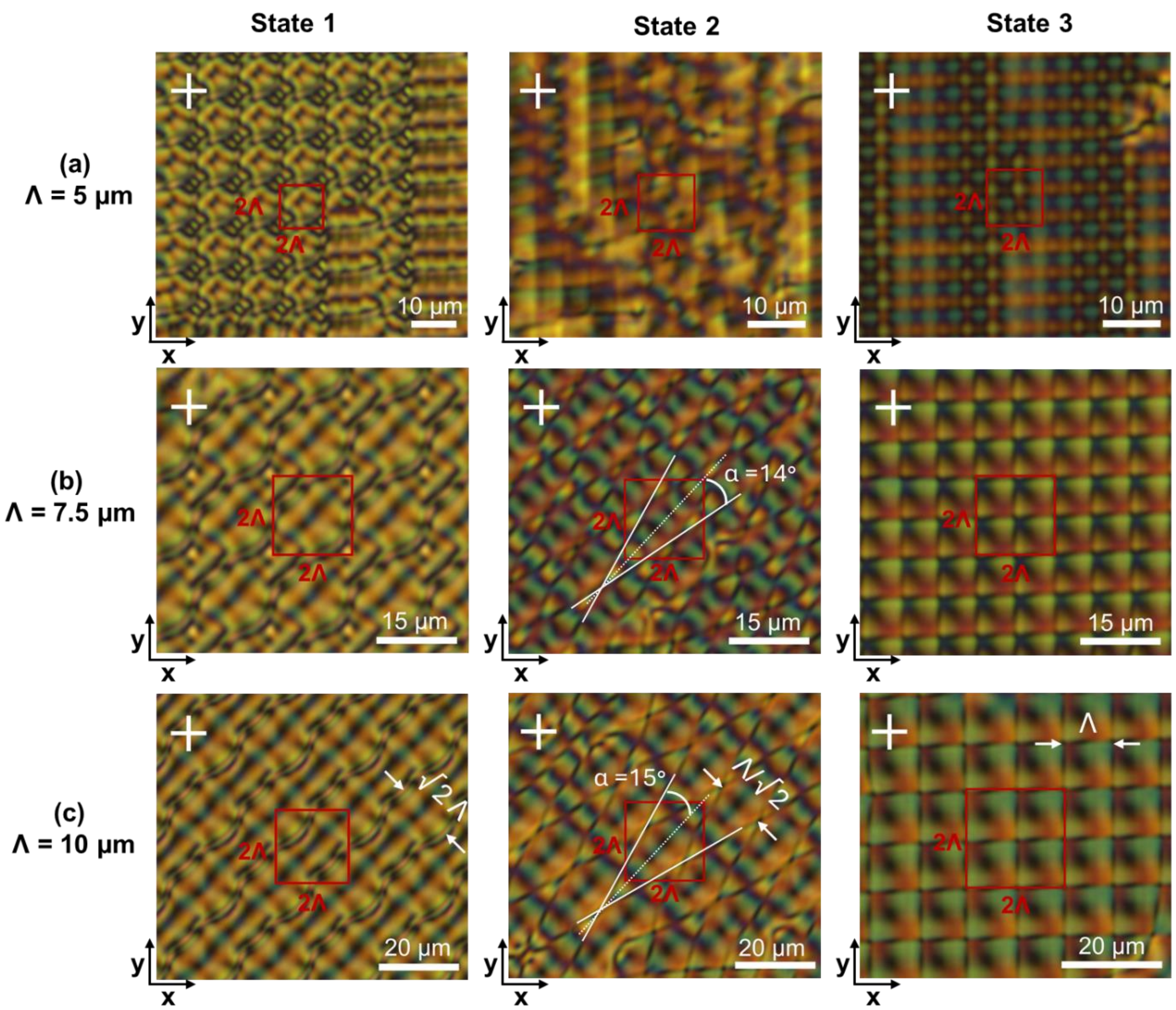

Figure S6. Experimental POM images of a 4.5 μm thick sample filled with DFLC 1952H. The three different states are shown for alignment period Λ = 5 μm (a), Λ = 7.5 μm (b) and Λ = 10 μm (c). The frequency and voltage paths used for switching between the different states are the same as shown in Figure 1(g-i) in the manuscript. Due to the pixelated nature of the SLM illumination used to produce this cell, the small alignment periods are less well resolved, giving rise to the formation of less uniform bulk textures. Also remark that a deformed state 2 is formed as well in the illuminated area with Λ = 10 μm (c), different from what is observed in the main manuscript for the alignment period Λ = 9.1 μm in a cell with d = 3.5 μm. The increased cell thickness (d = 4.5 μm) gives rise to the formation of a deformed state 2 also for larger alignment periods Λ. The same conclusion can be found by comparing Figure S8 and Figure S11 for cells filled with DFLC material 1999A. The measured value for α in the deformed state 2 is α ≈ 14° for Λ = 7.5 μm and 10 μm, similar to what is simulated in Figure 4(b) for the deformed state 2 in the 8Λ x 8Λ periodic unit cell.

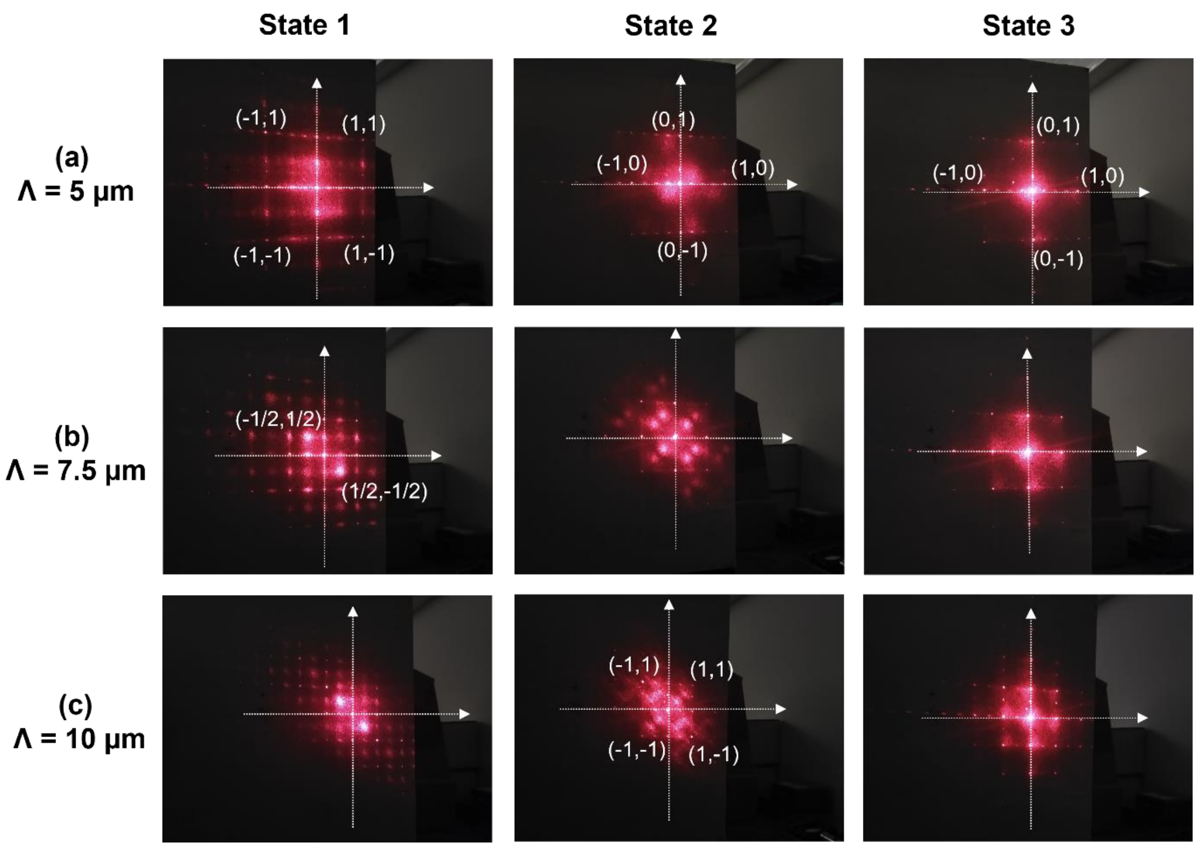

Figure S7. Experimental diffraction images of a 4.5 µm thick sample filled with DFLC 1952H. The three different states are shown for alignment period Λ = 5 µm (a), Λ = 7.5 µm (b) and Λ = 10 µm (c), corresponding with the POM images shown in Figure S6. The top substrate of the cell was oriented towards the incident red laser beam.

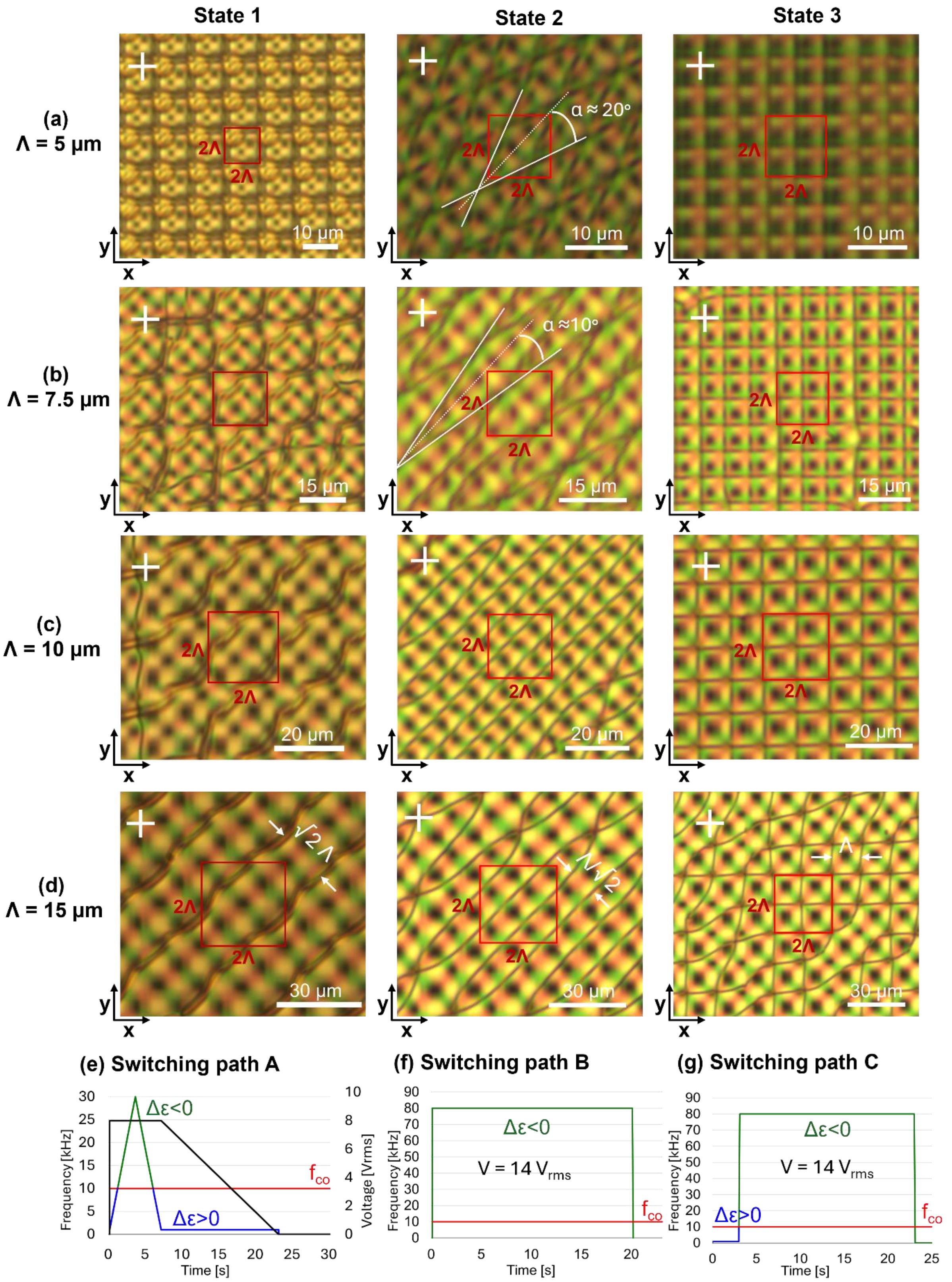

Figure S8. Experimental POM images of a 3.3 μm thick sample filled with DFLC 1999A. The three different states are shown with an alignment period Λ = 5 μm (a), Λ = 7.5 μm (b), Λ = 10 μm (c) and Λ = 15 μm (d). (e-g) frequency and voltage paths to reach each state following the same path definitions given in Figure 1(b). The green (blue) line indicates frequencies for which the material behaves negative (positive) anisotropic. The red line indicates the cross-over

frequency. The black line in (e) shows the change in voltage for path C while path A and B require a constant voltage of 14 V_{rms}. The measured value for α in the deformed state 2 is approximately $\alpha \approx 10°$ for $\Lambda = 7.5$ µm and $\alpha \approx 20°$ for $\Lambda = 5$ µm.

Switching path A: DFLC 1999A

Different paths were tested to optimise the uniformity of state 1 after switching. A few of these paths are visualised in supporting **Figure S9**. It was observed that simply applying a frequency in the positive dielectric regime is insufficient. This was the case for the regular voltage amplitude (8 V_{rms}, Figure S9 (a) and supporting **Video S8**) and for a twice larger voltage amplitude (16 V_{rms}, Figure S9 (b) and supporting **Video S9**). Crossing f_{co} once helps to destabilise the director configuration, which results in a more uniform state 1 (Figure S9 (c) and supporting **Video S10**), but decreasing the voltage steadily and combining it by crossing the f_{co} twice (Figure S9 (d) and supporting **Video S11** and Figure S9 (e) and supporting **Video S12**) did not substantially improve the uniformity of the state 1 configuration further. Path (e) was used during the acquisition of the experimental POM images while the other paths were examined later on during the research.

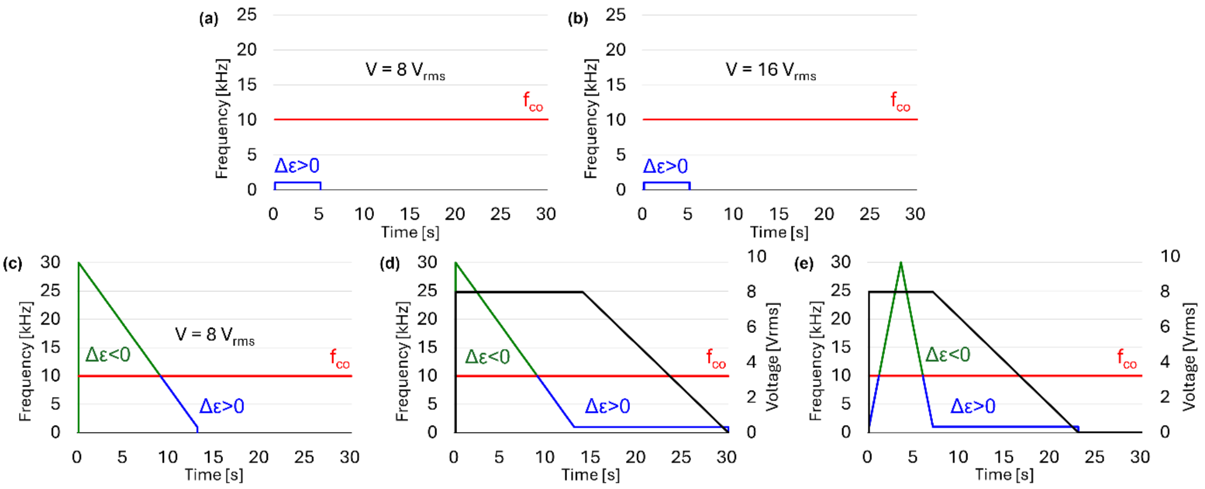

Figure S9. Different examined switching paths A for DFLC 1999A; (a) Applying a constant frequency of 1 kHz at 8 V_{rms} for 5 s (b) Applying a constant frequency of 1 kHz at 16 V_{rms} (c) Gradually decreasing the frequency and crossing the f_{co} from 30 kHz to 1 kHz at 8 V_{rms} and directly turning it off (d) Gradually decreasing the frequency and crossing f_{co} from 30 kHz to 1 kHz at 8 V_{rms} and reducing the voltage steadily to 0 V_{rms} (e) Crossing f_{co} twice by starting at 1 kHz, increasing the frequency to 30 kHz and reducing it back to 1 kHz. The voltage is then reduced steadily to 0 V_{rms}. The corresponding videos are shown in S8-S12.

Supplementary Video S8: this video shows the switching from state 3 to state 1 by applying a constant frequency of 1 kHz and a voltage of 8 V_{rms} for 5 seconds. This method is insufficient to reach a uniform state 3. The cell thickness is d = 5.4 µm, the DFLC material is 1999A and the video was made for alignment period Λ = 10 µm. The path is visualized in Figure S9(a).

Supplementary Video S9: this video shows the switching from state 3 to state 1 by applying a constant frequency of 1 kHz and a voltage of 16 V_{rms} for 5 seconds. This method is still insufficient to reach a uniform state 1, even after applying a much higher voltage. The cell thickness is d = 5.4 µm, the DFLC material is 1999A and the video was made for alignment period Λ = 10 µm. The path is visualized in Figure S9(b).

Supplementary Video S10: this video shows the switching from state 3 to state 1 by gradually reducing the frequency from 30 kHz to 1 kHz at a constant voltage of 8 V_{rms} and turning the voltage off directly. This path works to achieve a relatively uniform state 1. The cell thickness is d = 5.4 µm, the DFLC material is 1999A and the video was made for alignment period Λ = 10 µm. The path is visualized in Figure S9(c).

Supplementary Video S11: this video shows the switching from state 3 to state 1 by gradually reducing the frequency from 30 kHz to 1 kHz and gradually reducing the voltage afterwards. This path works to achieve a relatively uniform state 1, but does not improve further on the path shown in Figure S9(c) as only a statistically irrelevant deviating number of defects is achieved. The cell thickness is d = 5.4 µm, the DFLC material is 1999A and the video was made for alignment period Λ = 10 µm. The path is visualized in Figure S9(d).

Supplementary Video S12: this video shows the switching from state 3 to state 1 by crossing f_{co} twice by starting at 100 Hz, increasing the frequency gradually to 3kHz and reducing the frequency back to 100 Hz again while maintaining a constant voltage of 8 V_{rms}. The voltage is then gradually reduced. This path works to achieve a relatively uniform state 1, but does not improve further on the path shown in Figure S9(c). The cell thickness is d = 5.4 µm, the DFLC material is 1999A and the video was made for alignment period Λ = 10 µm. The path is visualized in Figure S9(e).

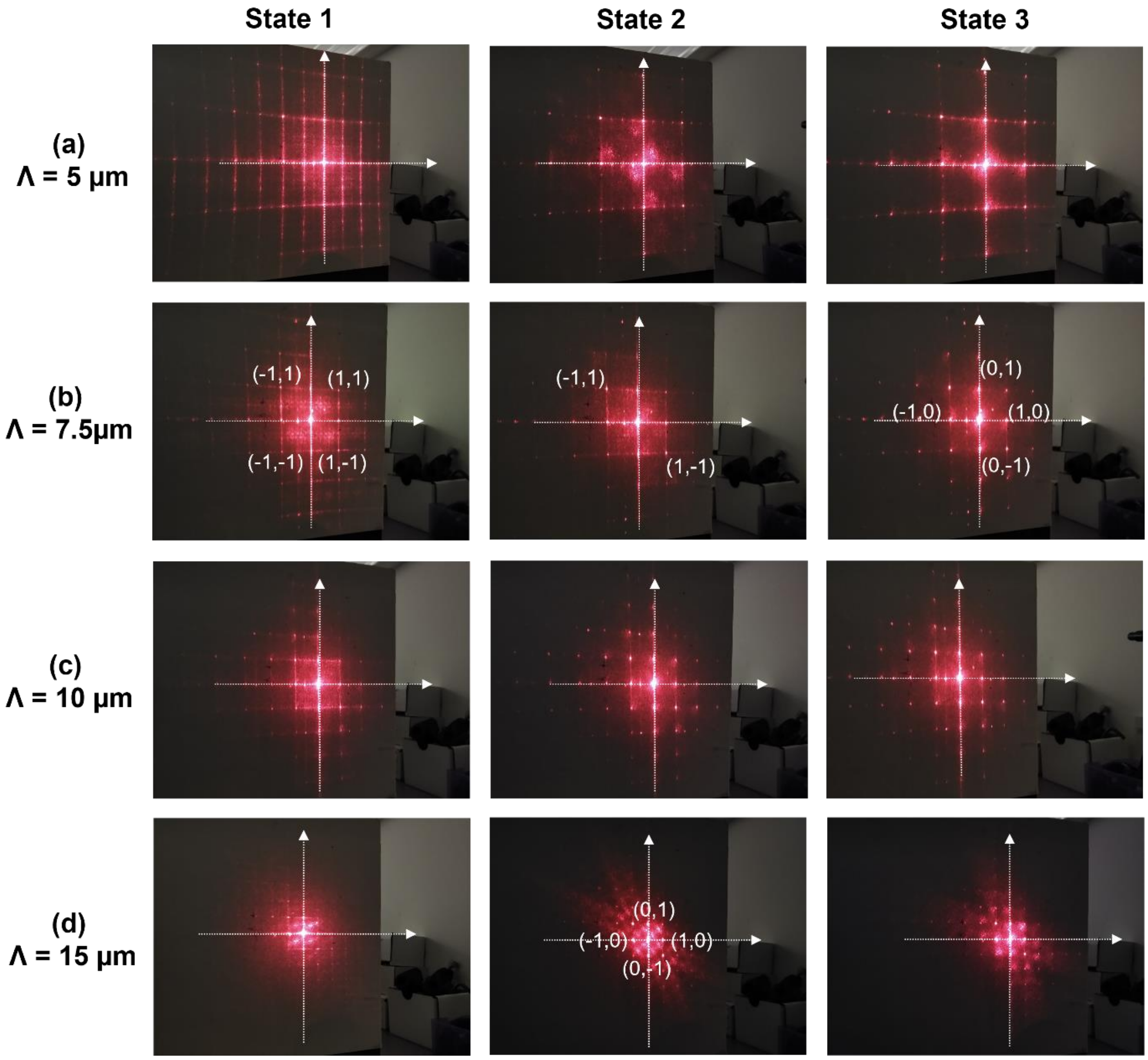

Figure S10. Experimental diffraction images of a 3.3 μm thick sample filled with DFLC 1999A. The three different states are shown with an alignment period Λ = 5 μm (a), Λ = 7.5 μm (b), Λ = 10 μm (c) and Λ = 15 μm (d), corresponding with the POM images shown in Figure S8. The top substrate of the cell was oriented towards the incident red laser beam.

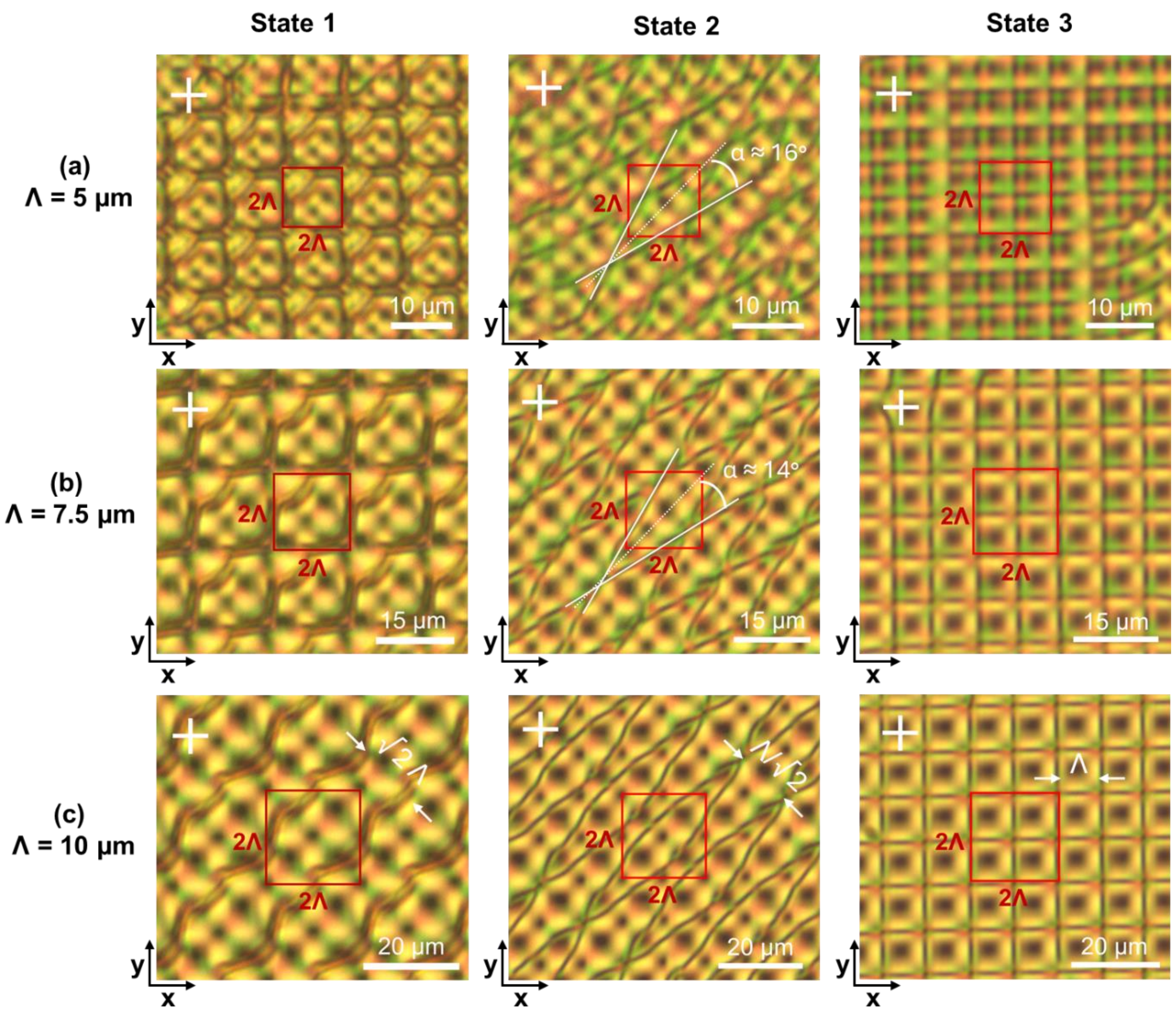

Figure S11. Experimental POM images of a 5.4 μm thick sample filled with DFLC 1999A. The three different states are shown with an alignment period Λ = 5 μm (a), Λ = 7.5 μm (b) and Λ = 10 μm (c). The switching paths are the same as the one shown in Figure S8(e-g). For this thicker cell (d = 5.4 μm compared to 3.3 μm in Figure S8), a deformed state 2 is also formed for the alignment period Λ = 10 μm, while the state 2 was only weakly deformed for Λ = 10 μm and Λ = 15 μm in the 3.3 μm thick cell (Figure S8). Thicker cells tend to form a deformed state 2 somewhat more easily, as can also be observed by comparing Figure S6 with Figure 1 in the main manuscript for the DFLC 1952H. The measured value for α in the deformed state 2 is approximately α ≈ 14° for Λ = 7.5 μm and α ≈ 16° for Λ = 5 μm, roughly corresponding to what is simulated in Figure 4(b) for the deformed state 2 in the 8Λ x 8Λ periodic unit cell.

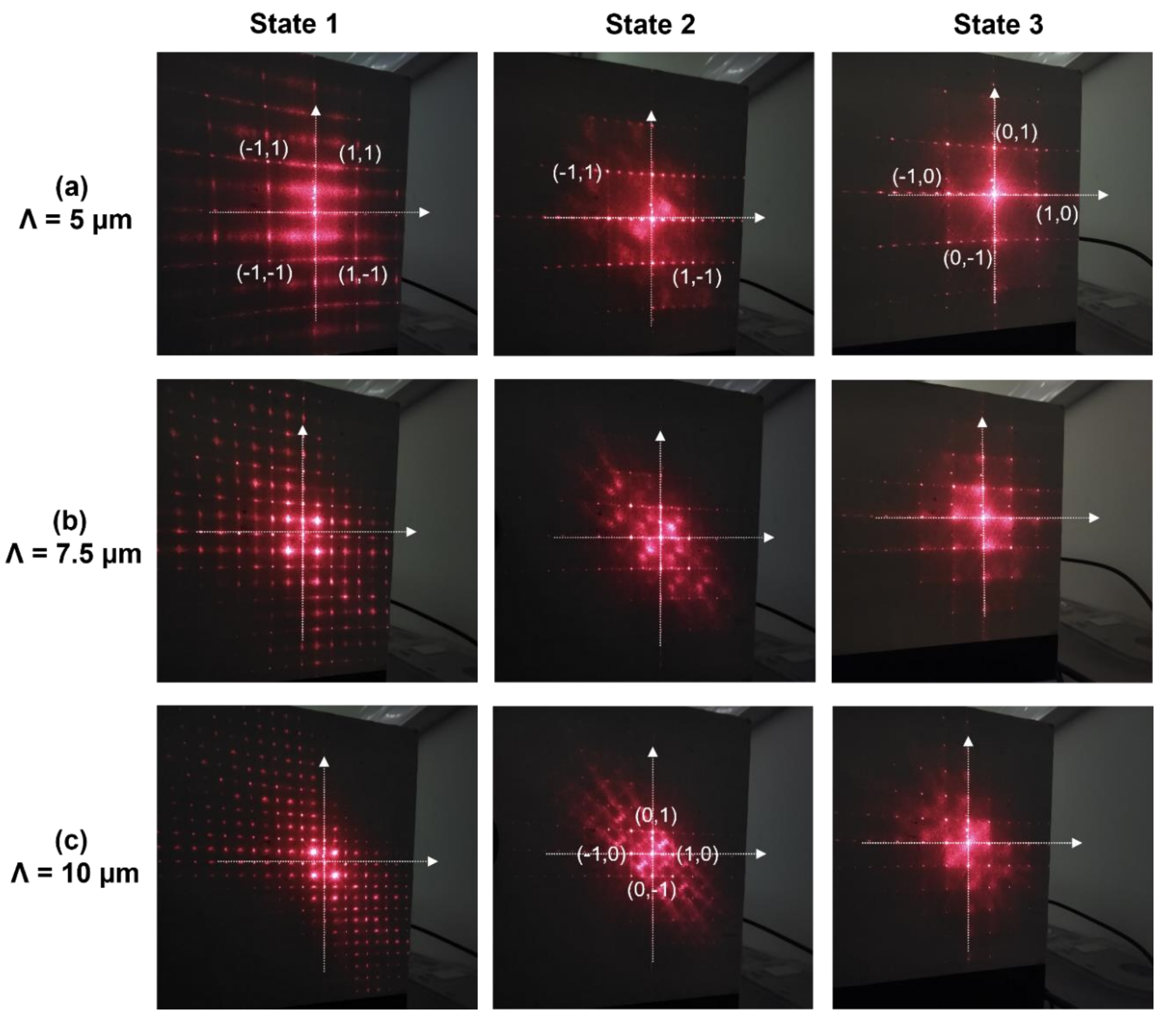

Figure S12. Experimental diffraction images of a 5.4 µm thick sample filled with 1999A. The three different states are shown with an alignment period Λ = 5 µm (a), Λ = 7.5 µm (b) and Λ = 10 µm (c) , corresponding with the POM images shown in Figure S11. The top substrate of the cell was oriented towards the incident red laser beam.

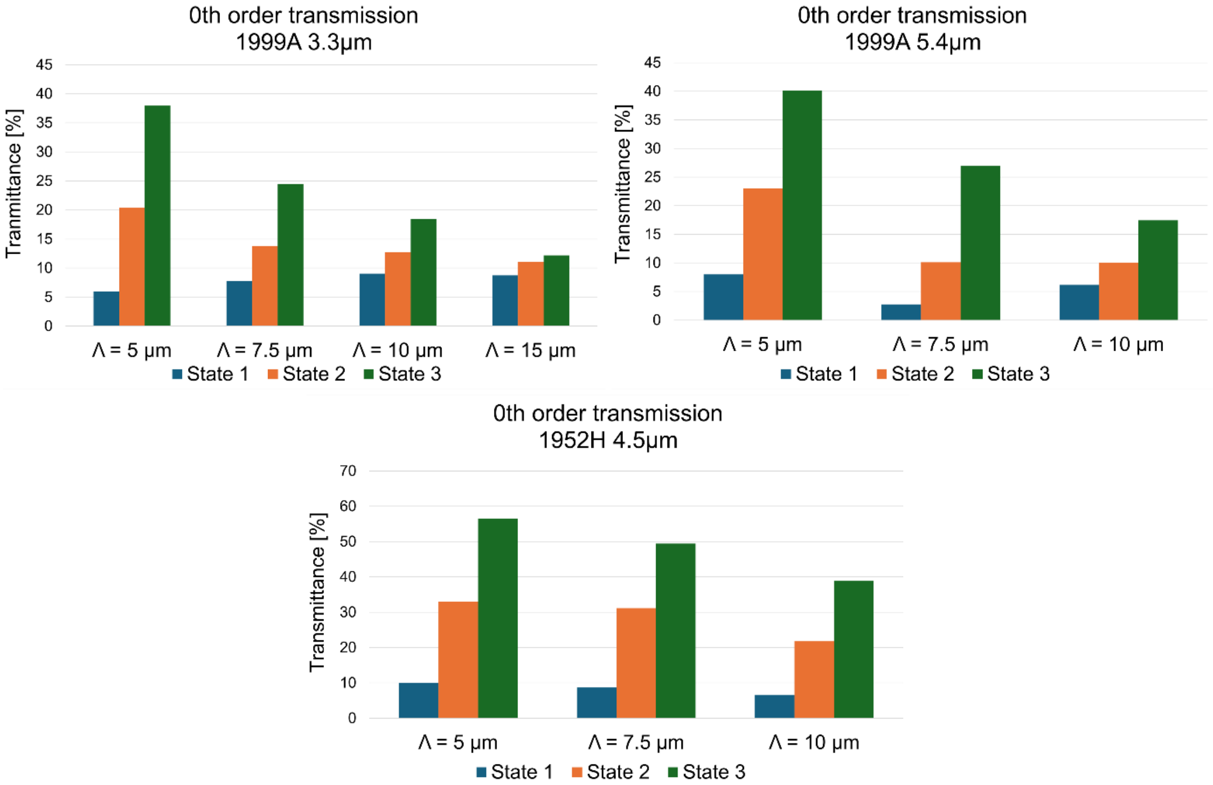

Figure S13. Experimentally measured zero order diffraction efficiency for two samples filled with 1999A DFLC material, one with *d* = 3.3 μm (a) and the second with *d* = 5.4μm (b), and another sample filled with 1952H DFLC material with *d* = 4.5 μm thickness (c). The sample with 1952H DFLC material that is presented in the manuscript has a thickness *d* = 3.5 μm.

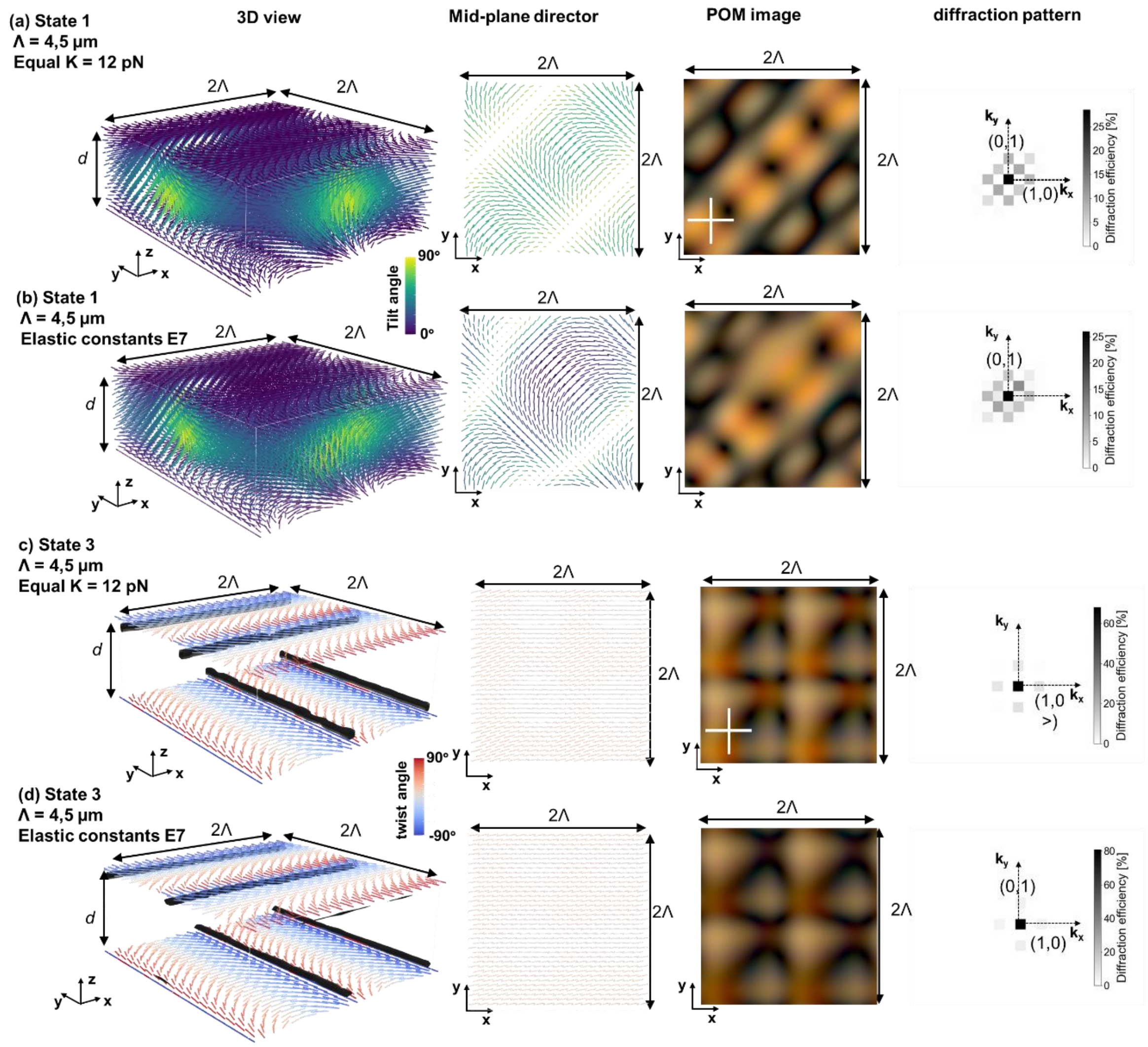

Figure S14. Simulated director configuration, POM image and diffraction pattern in state 1 (a,b) and state 3 (c,d) without applied voltage in a cell with thickness d = 3.5 µm and alignment period Λ = 4.5 µm. The results in (a,c) are obtained by using equal elastic constants $K_{11} = K_{22} = K_{33}$ = 12 pN in the simulations while the elastic constants of E7 (K_{11} = 11.1 pN , K_{11} = 6.5 pN , K_{33} = 17.1 pN) are used in (b,d). From left to right a 3D view, the mid-plane cross-section, the simulated POM image and the simulated diffraction pattern for red light λ = 633nm are shown. The color bar represents the tilt-angle with respect to the xy-plane.

Figure S14 compares the simulated director configuration, POM image and diffraction pattern for a case in which equal elastic constants $K_{11} = K_{22} = K_{33}$ = 12 pN are used (a,c) and a case in which the elastic constants of E7 are used K_{11} = 11.1 pN , K_{11} = 6.5 pN , K_{33} = 17.1 pN (b,d). The (roughly diagonal) lines with vertical mid-plane director orientation in state 1 (Figure S14 (a,b)) are straight in the case of equal elastic constants (a) but start to bend for unequal elastic

constants (b). This is accompanied by variations of the tilt-angle observed in the intermediate regions with close to planar mid-plane director orientation in the case of unequal elastic constants (b). The apparent symmetry in the resulting POM image is broken when unequal elastic constants are used and this effect becomes more pronounced for decreasing alignment periods.[1,2] The distribution of energy in the different diffraction orders is also slightly influenced by the exact values of the elastic constants, although the effect is not very pronounced for the chosen examples (with respectively equal K = 12 pN and the elastic constants of E7). The simulations for state 3 (Figure S14 (c,d)) demonstrate that the same topological configuration, consisting of a grid of ½ strength twist-disclination lines can be obtained irrespective of the values of the elastic constants. The exact director configuration and diffraction pattern in state 3 is influenced by the exact values of the elastic constants, but the effect is not very pronounced for the chosen examples (with respectively equal K = 12 pN and the elastic constants of E7).

References

[1] I. Nys, J. Beeckman, K. Neyts, *Soft Matter* **2015**, *11*, 7802-7808.
[2] I. Nys, V. Nersesyan, J. Beeckman, K. Neyts, *Soft Matter* **2018**, *14*, 6892–6902.
[3] V. Nersesyan, I. Nys, F. Van Acker, C.-T. Wang, J. Beeckman, K. Neyts, *J. Mol. Liq.* **2020**, *306*, 112864.
[4] I. Nys, B. Berteloot, K. Neyts, *J. Mol. Liq.* **2023**, *386*, 122472.
[5] B. Berteloot, I. Nys, G. Poy, J. Beeckman, K. Neyts, *Soft Matter* **2020**, *16*, 4999–5008.
[6] I. Nys, B. Berteloot, G. Poy, *Crystals* **2020**, *10*, 840.
[7] I. Nys, P. Ropac, B. Berteloot, M. Ravnik, K. Neyts, *J. Mol. Liq.* **2023**, *383*, 122062.
[8] R. Mazur, W. Piecek, Z. Raszewski, P. Morawiak, K. Garbat, O. Chojnowska, M. Mrukiewicz, M. Olifierczuk, J. Kedzierski, R. Dabrowski, D. Weglowska, *Liquid crystals.* **2016**, *44(2)*, 1216621.
